# The spatial distribution of a hummingbird‐pollinated plant is not strongly influenced by hummingbird abundance

**DOI:** 10.1002/ajb2.70034

**Published:** 2025-04-26

**Authors:** Matthew L. Coffey, Andrew M. Simons

**Affiliations:** ^1^ Department of Biology Carleton University 1125 Colonel By Drive Ottawa K1S 5B6 Canada

**Keywords:** *Archilochus colubris*, community science, *Lobelia cardinalis*, MaxEnt, plant‐pollinator mutualism, pollinator abundance, specialized pollination, species distribution

## Abstract

**Premise:**

Many angiosperms have evolved specialized systems that promote pollination by specific taxa. Therefore, plant distributions may be limited by the local abundance of their specialist pollinators. In eastern North America, *Lobelia cardinalis* is thought to be pollinated solely by *Archilochus colubris*, the only hummingbird species found in the region. Here we tested the hypothesis that the distribution of a plant species with specialized pollination is controlled by the range and abundance of its specialist pollinator.

**Methods:**

We investigated the importance of *A. colubris* abundance, sourced from eBird, as a variable in a MaxEnt species distribution model of *L. cardinalis* using presence data from iNaturalist. We also compared hummingbird abundance between locations of *L. cardinalis* and congeneric during their respective flowering periods and explored whether the flowering periods of *L. cardinalis* and congenerics align with the week of peak local hummingbird abundance.

**Results:**

Unexpectedly, MaxEnt modelling did not suggest that *A. colubris* abundance is a key driver of the species distribution. *Lobelia cardinalis* habitat suitability was lowest in the absence of *A. colubris* and increased with increasing abundance, but habitat suitability was also low in regions where hummingbird abundance is highest. Still, hummingbird abundance at *L. cardinalis* locations was generally higher than most congenerics, and *L. cardinalis* tended to flower near the week of local peak *A. colubris* abundance.

**Conclusions:**

While populations of hummingbird‐pollinated plant species may require the local presence of hummingbirds, fine‐scale variation in hummingbird abundance may not strongly influence their spatial distributions.

Plant–pollinator relationships have influenced diversification and biodiversity at the macroevolutionary scale (Stephens et al., 2023), and these relationships—particularly specialization—have long been a central focus in evolutionary ecology and natural history (Sprengel, 1793; Darwin, 1859, 1876; Muller, 1883; Knuth, 1908, 1909; Baker, 1963; Grant and Grant, 1965; Faegri and van der Pijl, 1979). Most angiosperm pollination occurs through animal vectors like insects, birds, and bats, with specialized pollination defined as pollination by a relatively small number of species or functional groups of species (Ollerton et al., 2011; Armbruster, 2017). When plant species specialize to a small subset of pollinators, the non‐uniform distribution of these pollinators may affect plant geographic distributions.

Specialized pollination may also limit species distributions. Many species distribution limits occur along continuous abiotic environmental gradients where conditions past the limit are thought to determine regions where populations can no longer persist (Brown et al., 1996; Gaston, 2003; Holt, 2003; Sagarin et al., 2006). Consequently, traditional species distribution modelling efforts typically focus on the importance of abiotic factors like temperature and precipitation (Sexton et al., 2009; Bradie and Leung, 2017). However, empirical studies have begun to challenge the traditional niche limit view because many plant species do not exhibit lower fitness in peripheral populations (González‐Guzmán and Mehlman, 2001; Gilman, 2006; Sagarin et al., 2006; Samis and Eckert, 2007, 2009). Many species even maintain sustainable populations when planted beyond their traditional range limits (Van Der Veken et al., 2007; Samis and Eckert, 2009; Samis et al., 2016) with some populations even showing no evidence of local adaptation to beyond‐range environments (Cross and Eckert, 2024). Therefore, some species distributions are likely limited by dispersal or unstable metapopulation dynamics rather than, or in addition to, the traditional niche limits (Gaylord and Gaines, 2000; Holt and Keitt, 2000; Holt et al., 2005). Also overlooked by traditional species distribution modelling are biotic factors including interspecific interactions, likely because they can be difficult to quantify. However, the omission of interspecific interactions from models may be important because species distributions are known to be limited by factors such as competition (Bullock et al., 2000; Armitage and Jones, 2020) and plant–pollinator mutualisms (Chalcoff et al., 2012; Wisz et al., 2013).

Variable pollination conditions (e.g., insufficient pollen deposition, pollen limitation, variation in pollinator abundance, and reliability over both short‐ and long timescales) can have significant consequences on sexual reproductive success and, as a result, individual fitness, population demography, and genetic diversity (Burd, 1994; Larson and Barrett, 2000; Ashman et al., 2004; Knight et al., 2005; Aizen and Harder, 2007; Thomson, 2019). For example, short‐term temporal variation in pollinator abundance can affect mating systems through the evolution of risk reduction or conservative bet hedging in fundamental fitness characters such as increased ovule number per flower (Burd et al., 2009; Simons, 2011). Continual pollen limitation is also predicted to select for increased self‐compatibility in plant species as a mode of reproductive assurance when conspecifics are rare and/or when pollinator service is unreliable (Lloyd, 1980; Schoen and Brown, 1991; Morgan and Wilson, 2005; Porcher and Lande, 2005). Consequently, range margins are often associated with increased self‐compatibility, floral traits associated with selfing, and generally higher rates of self‐fertilization (Ledig et al., 2005; Darling et al., 2008; Griffin and Willi, 2014; Coffey and Simons, 2023). However, in plant species that suffer high inbreeding depression (fitness costs resulting from increased expression of deleterious recessive alleles; Jain, 1976; Charlesworth and Charlesworth, 1987; Charlesworth and Willis, 2009), the impacts of pollen limitation due to unreliable pollination may be more pronounced. Therefore, we expect the distributions of such species to be strongly controlled by the geographic range of their specialized pollinators.

Cardinal flower (*Lobelia cardinalis* L.; Campanulaceae) provides an intriguing case study for the importance of plant–pollinator interactions on species distributions. *Lobelia cardinalis* is an angiosperm long purported to be solely pollinated in the eastern part of its range by the Ruby‐Throated Hummingbird (*Archilochus Colubris*), the only hummingbird species (Trochilidae) in eastern North America (Bertin, 1982; Devlin and Stephenson, 1984, 1985; Devlin et al., 1987; Bartkowska and Johnston, 2014). However, the extent to which species‐level pollinator specialization has impacted the geographic distribution of *L. cardinalis* has not been well studied. Cardinal flower is known to have protandrous flowers (male phase first), with no overlap between sexual phases (thus obligately outcrossing), and suffer high rates of inbreeding depression (Devlin and Stephenson, 1984; Johnston, 1992). Therefore, the distribution of *L. cardinalis* should be strongly controlled by the distribution and abundance of its pollinator, *A. colubris*, because locally abundant pollinators are expected to increase outcrossing rates and the absence of pollinators should act as a range limit—failure to outcross confers a fitness detriment. Conversely, since *A. colubris* does not solely forage on *L. cardinalis* (Bertin, 1982), the distribution of *A. colubris* is not expected to be strongly influenced by the distribution of *L. cardinalis*.

Here we tested the hypothesis that the distribution of an outcrossing plant species with specialized pollination is controlled by the range and abundance of its pollinators. We explored the importance of the local abundance of *A. colubris*, sourced from eBird's Status and Trends Data (Fink et al., 2023), as a variable in a maximum entropy (MaxEnt) species distribution model of *L. cardinalis* (also referred to as an ecological niche model; Phillips et al., 2006). We predicted that *A. colubris* abundance will be an important variable in the MaxEnt model and that high abundance will be associated with greater *L. cardinalis* habitat suitability.

Because pollinator abundance has not previously been included as a factor in MaxEnt models, we can make no a priori predictions about the relative strength of contribution of *A. colubris* abundance compared to abiotic factors such as temperature that are normally found to contribute strongly to species distributions. Therefore, we subjected the main hypothesis to two further tests. First, we compared *A. colubris* abundance between presence locations of *L. cardinalis* and locations of congeneric *Lobelia* species. Unlike *L. cardinalis*, congeneric *Lobelia* species in eastern North America are insect‐pollinated or highly selfing (Caruso and Case, 2007; Hughes and Simons, 2015; Coffey and Simons, 2023); thus, we predicted higher *A. colubris* abundance where *L. cardinalis* occurs. Second, we explored the extent to which the flowering periods of *L. cardinalis* and congeneric *Lobelia* species line up with the week of peak local *A. colubris* abundance. We predicted that flowering observations of *L. cardinalis*, and not necessarily those of congeneric species, will occur close to the week of peak local *A. colubris* abundance.

## MATERIALS AND METHODS

All data analyses were performed using R version 4.4.1 (R Core Team, 2024). Data and map visualizations were built using R packages ggplot2, terra, and tidyterra (Wickham, 2016; Hernangómez, 2023; Hijmans, 2023).

### *L. cardinalis* occurrence data

Presence records for *L. cardinalis* were sourced from the iNaturalist Research Grade Observations data set through the Global Biodiversity Information Facility (GBIF.org, 2024a). Because the eBird Status and Trends Data contain weekly bird abundance models for the 2022 calendar year, we chose to restrict our *L. cardinalis* presence records to only include observations made in 2022, in the United States and Canada, and with a maximum coordinate uncertainty of 100 m (*N* = 2286; Fink et al., 2023; GBIF.org, 2024a). Although restricting presence points to a single year ignores among‐year variation in *L. cardinalis* presence locations, corresponding among‐year variation in hummingbird abundance is not captured by the eBird models. Therefore, we felt it most appropriate to match the year of the *L. cardinalis* presences to the year of the eBird models. Presence records were clipped to an arbitrarily defined boundary for eastern North America (–100° to –50° longitude, 24.53° to 60° latitude) and spatially thinned to ensure one presence record per environmental raster cell (1‐km spatial resolution; *N* = 1850). After we removed presence records that fell into a raster cell that lacked any environmental data, 1747 presence records remained (Appendix S1: Figure S1).

### Environmental variables

MaxEnt builds a model, using species presence records, with a set of environmental variables representing factors that could influence the environmental suitability for a particular species (Phillips et al., 2006). Climate, soil, terrain, and land cover are known to affect plant distributions and are commonly important variables in MaxEnt models (Bradie and Leung, 2017; Chauvier et al., 2021; Huang et al., 2024). We used a diverse set of environmental variables in modelling including 19 bioclimatic variables, nine soil variables, six landcover variables, one terrain variable, and one pollinator abundance variable (Table 1). These environmental variables (except for pollinator abundance) were sourced using the R package geodata (Hijmans et al., 2023a) at 1‐km spatial resolution; these variables and their sources are detailed in Table 2. To best quantify the effect of *A. colubris* abundance on the distribution of *L. cardinalis*, we used the average abundance of *A. colubris* during flowering of *L. cardinalis*, defined as 2 SD around the mean iNaturalist observation date of all the presence records (1 July to 13 October; Appendix S1: Figure S2; Appendix S2: Table S1). For each week during this period (15 weeks), we acquired a raster map layer (3‐km resolution) of the median weekly *A. colubris* abundance from eBird Status and Trends Data Products (using R package ebirdst; Strimas‐Mackey et al., 2023). The eBird models define abundance as the count of individuals of a particular species detected by an expert eBirder over a 1‐h, 2‐km traveling checklist at the optimal time of day (Fink et al., 2023). We then averaged the 15 weekly rasters to get a single raster, which was then bilinearly resampled to 1‐km resolution (Appendix S1: Figure S2). All rasters were cropped to the same extent as the above presence locations for *L. cardinalis* and masked to ensure missing values were shared between environmental raster layers. We accounted for multicollinearity among environmental variables using Pearson's correlation coefficient.When two environmental variables were correlated at |*r*| > 0.8, we chose the more fundamental variable for use in modelling (e.g., annual precipitation and precipitation of the coldest quarter were highly correlated, so the former was selected). After variable selection, the number of environmental variables used in final modelling was reduced from 36 to 16 (see Table 1; Appendix S1: Figure S4).

**Table 1:** List of candidate environmental variables. Right‐most column indicates whether the variable was chosen to be used in MaxEnt modelling following analysis of pairwise correlation coefficients.

Type	Description	Unit	Source	Reference	Used in model
Pollinator	Mean abundance of *A. colubris*	—	eBird	Fink et al. (2023)	*✓*
Landcover	Tree cover fraction	%	ESA Worldcover	Zanaga et al. (2021)	*✓*
	Cropland cover fraction	%			*✓*
	Water cover fraction	%			*✓*
	Grassland cover fraction	%			*✓*
	Shrubland cover fraction	%			*✓*
	Wetland cover fraction	%			*✓*
Terrain	Elevation	m	SRTM	Jarvis et al. (2008)	*✓*
Soil	Bulk density of fine earth fraction	kg·dm^−3^	SoilGRIDS	Poggio et al. (2021)	
	Volume fraction of coarse fragments (>2mm)	%			
	Total nitrogen (N)	g·kg^−1^			*✓*
	pH (H_2_O)	—			*✓*
	Sand (>0.05 mm) in fine earth	%			*✓*
	Silt (0.002–0.05 mm) in fine earth	%			
	Clay (<0.002 mm) in fine earth	%			
	Soil organic carbon in fine earth	g·kg^−1^			*✓*
	Organic carbon density	kg·m^−3^			
Climate	Mean annual temperature	°C	WorldClim	Fick and Hijmans (2017)	*✓*
	Mean diurnal range	°C			*✓*
	Isothermality	%			
	Temperature seasonality	%			
	Max temperature of warmest month	°C			
	Min temperature of coldest month	°C			
	Temperature annual range	°C			
	Mean temperature wettest quarter	°C			*✓*
	Mean temperature driest quarter	°C			
	Mean temperature warmest quarter	°C			
	Mean temperature coldest quarter	°C			
	Annual precipitation	mm·m^−2^			*✓*
	Precipitation wettest month	mm·m^−2^			
	Precipitation driest month	mm·m^−2^			
	Precipitation seasonality	%			
	Precipitation wettest quarter	mm·m^−2^			
	Precipitation driest quarter	mm·m^−2^			
	Precipitation warmest quarter	mm·m^−2^			
	Precipitation coldest quarter	mm·m^−2^			

**Table 2:** Percent contribution (PC) and permutation importance (PI) of each environmental variable for the final MaxEnt model. Variables are ordered from highest to lowest percent contribution.

Variable	PC (%)	PI (%)
Cropland cover	32.78	34.60
Water cover	29.93	14.98
Tree cover	13.62	11.16
Mean annual temperature	8.50	12.74
Grassland cover	6.69	13.81
Elevation	3.16	4.90
*A. colubris* abundance	1.59	0.37
Soil pH	1.20	3.76
Soil organic carbon	1.17	1.49
Sand	0.68	1.11
Wetland cover	0.55	0.78
Annual precipitation	0.07	0.03
Mean diurnal range	0.05	0.15
Shrubland cover	0.02	0.13
Mean temperature wet quarter	0.00	0.00
Nitrogen	0.00	0.00

### Species distribution modelling

#### Background points

MaxEnt is a presence‐only ecological niche model, so a random sample of background points (here, 10,000) are used as pseudo‐absences (Phillips et al., 2006; Phillips and Dudík, 2008). Since community science observations are typically biased toward roads, human population centers, and protected areas (Geurts et al., 2023), we used a target‐group bias‐corrected background, an effective method of correcting for spatial biases in presence data (Phillips and Dudík, 2008; Phillips et al., 2009; Barber et al., 2022). Background points were manipulated so they share the same biases as the presences by quantifying and applying the relative sampling effort of a target group (Barber et al., 2022), which, in our case, was Campanulaceae. We manipulated the random sampling of background points using a weighted bias file—a 2D kernel density estimate of the locations of all research grade iNaturalist observations made of Campanulaceae species in the United States and Canada since iNaturalists inception (101,748 observations; GBIF.org, 2024b)—to produce a set of 10,000 background points that share the same spatial biases as the presence data (Appendix S1: Figure S1).

#### Model optimization

Although default MaxEnt parameters are often used to build models, this strategy may result in overly complex models prone to overfitting, making results difficult to interpret (Warren and Seifert, 2011; Radosavljevic and Anderson, 2014; Warren et al., 2014). We used the R package ENMeval (Muscarella et al., 2014) to optimize our model by evaluating different combinations of two model parameters: feature classes, which indicate the type/shape of curves fit to the environmental variables, and the regularization multiplier, which indicates the how much model complexity (i.e., number of coefficients) should be penalized (Elith et al., 2011). We selected four potential feature classes, L (linear), LQ (linear and quadratic), H (hinge), and LQH (linear, quadratic, and hinge) and varied the regularization multiplier from 1 to 5 in intervals of 0.5, resulting in 36 potential combinations. This set of feature classes was selected because they produce the simplest response curves, which is useful when interest lies in predictor importance (Merow et al., 2013). All 36 parameter combinations were evaluated using the corrected Akaike information criterion (AICc), and the combination with the lowest AICc was chosen as the optimal parameter set (Muscarella et al., 2014). For our model, the parameter combination with the lowest AICc included linear, quadratic, and hinge features (LQH) and a regularization multiplier of 3 (see Appendix S2: Table S2).

#### Model training and evaluation

**Figure 1:**
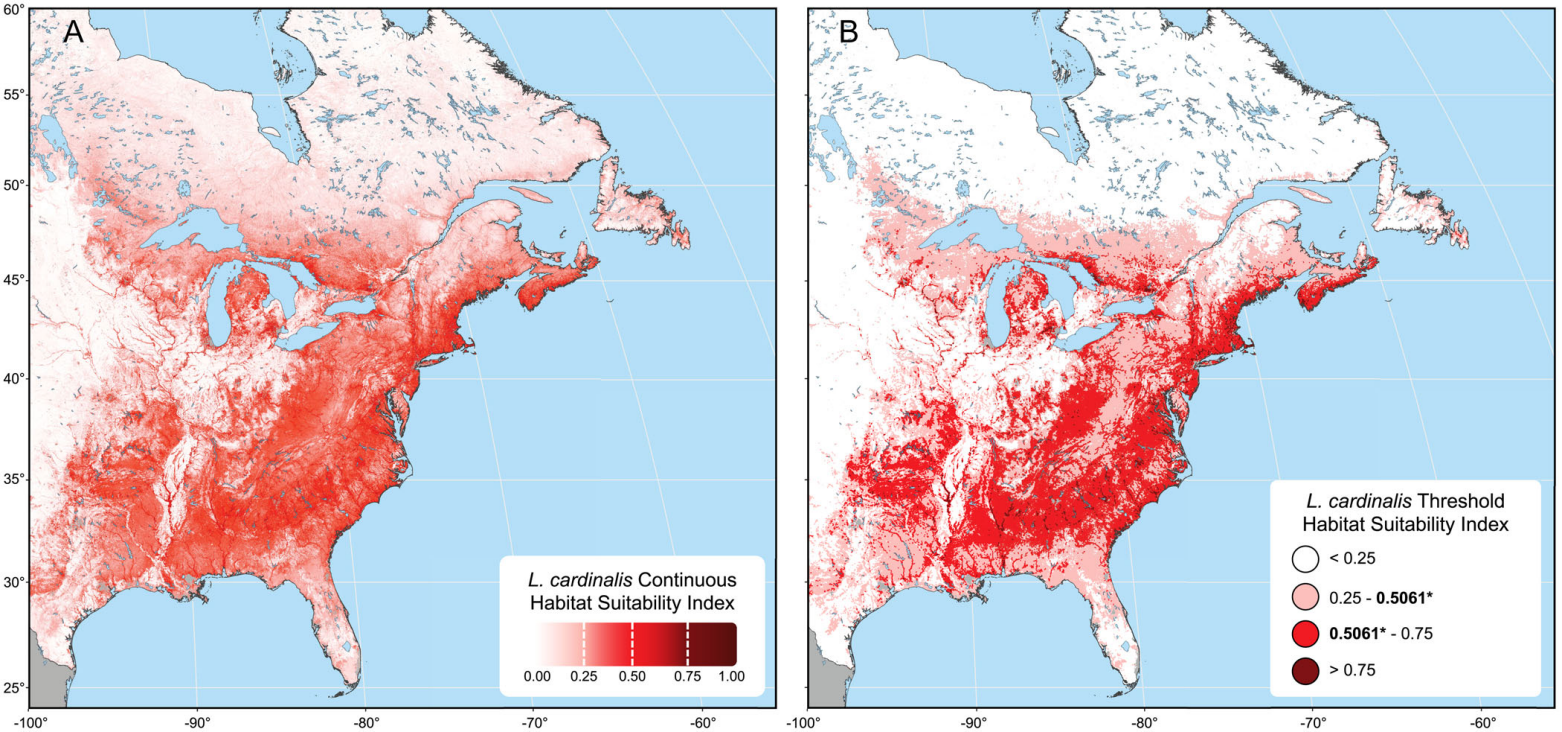
(A) Continuous and (B) threshold habitat suitability map predictions for *Lobelia cardinalis*. Predictions are based on the final MaxEnt model. In (A), habitat suitability is presented as a continuous metric bounded between 0 and 1 with darker areas showing regions of the highest habitat suitability. In (B), the continuous metric has been binned into categories based on the maxSSS cloglog threshold (0.5061) with any regions shown in red or deep red indicating habitat suitability above the threshold and, as a result, likely presence of *L. cardinalis*. Regions outside the model study area are in grey. Maps were created using a Mollweide equal‐area projection with a central meridian at –100° longitude.

The optimized MaxEnt model was fit using the maxent function in MaxEnt version 3.4.3 (R package dismo; Hijmans et al., 2023b), with cloglog output, which creates a continuous habitat suitability index scaled between 0 and 1 (see Figure 1A). To cross‐validate model building, we trained a model on 80% of the presence records and withheld 20% to serve as testing data. Model performance was then evaluated for both the training and testing data using two methods: the area under the receiver operating curve (AUC) and continuous Boyce index (CBI). The AUC values, calculated using the function evaluate (R package dismo; Hijmans et al., 2023b), vary from 0 to 1 and indicate the model's ability to distinguish presence points from background points; values above 0.7 are considered fair model performance (Phillips et al., 2006). The CBI values, calculated using the function Boyce (R package modEvA; Márcia Barbosa et al., 2013), vary from –1 to 1 and quantify the model's ability to predict the presence data alone; values closer to 1 indicate greater model effectiveness (Boyce et al., 2002; Hirzel et al., 2006). If the training and testing AUC and CBI were above thresholds of 0.7 and 0.5, respectively, we built a final model using all 1747 presence records as the training data.

To account for temporal variation in the presence locations for *L. cardinalis* across years and/or possible iNaturalist sampling error in the 2022 data, we evaluated our final MaxEnt model's effectiveness at predicting iNaturalist observations of *L. cardinalis* made in 2023 through both AUC and CBI (GBIF.org, 2024c). For consistency, the 2023 presence locations were subjected to the same filtering as the 2022 data (see above), which resulted in 1844 presence locations for use in model evaluation. In addition, to confirm whether multiple years of *L. cardinalis* presence data would strongly influence model building, we also built a supplementary model (Appendix S1: Figures S7, S8; Appendix S2: Tables S6, S7) using presence data from 2018 to 2022 (5432 presence points used in final model building; GBIF.org, 2025). We found no strong differences between this supplementary model and our final model, which used only the 2022 presence data, providing further validation for the use of presence data from a single year.

#### Thresholding

MaxEnt provides several cloglog thresholds, defined based on model training, with which the continuous habitat suitability metric can be converted into a binary measure of likely presence and absence of the focal species. We used the maximum sum of sensitivity and specificity threshold (maxSSS), previously shown to be an effective threshold for presence‐only data, to convert our continuous habitat suitability into binary metric indicating likely presence or absence of *L. cardinalis* (Liu et al., 2005, 2016; see Figure 1B).

#### Contribution of environmental variables

To quantify the effect and contribution of *A. colubris* abundance and the other environmental variables to the model, we used three metrics of variable contribution (percent contribution, permutation importance, and jackknife testing) and evaluated the shapes of the environmental variable response curves. Percent contribution indicates how much each environmental variable contributes to the final model based on the effect of adjusting the variable's coefficient during model training. Permutation importance refers to the sensitivity of the model prediction to changes in a particular environmental variable—i.e., the increase in prediction error from a random permutation of the values of a variable (Phillips et al., 2006). For the jackknife test, two additional models are produced for each environmental variable: one where the variable is excluded from model building and another where the variable is used in isolation. The regularized training gain of these two additional models (with and without each variable) is calculated and then compared to the gain of the full model.

MaxEnt produces two different response curves that show how habitat suitability for *L. cardinalis* varies with the magnitude of each environmental variable. The first is a marginal response curve showing variation in habitat suitability when all other environmental variables are held at their mean value. The second response curve, which is often more easily interpreted given correlations among predictors, is based on the model where the environmental variable was used in isolation. We examined the shape of each curve and assessed where it exceeded the maxSSS threshold, indicating the range of environmental values associated with likely *L. cardinalis* presence.

### Congeneric species comparisons

We compared the local *A. colubris* abundance between presence locations of *L. cardinalis* and congenerics, none of which are hummingbird‐pollinated. We downloaded a data set from GBIF of all research grade iNaturalist observations of *Lobelia* species made in the United States and Canada in 2022 (*N* = 27 species; GBIF.org 2024a) and filtered the records under the same specifications as described above for *L. cardinalis*. The data set was also filtered to only include species with 100 or more records, resulting in six species (including *L. cardinalis*) for use in analysis (*N* = 4663 presence records; see Appendix S2: Table S1).

We performed two analyses to explore differences in local *A. colubris* abundance between *Lobelia* species. First, we asked whether the local abundance of *A. colubris* differed during the flowering seasons of *L. cardinalis* and congenerics. For each *Lobelia* species, we used 2 SD around the mean observation date as an estimate of each species' flowering period (Appendix S1: Figure S2; Appendix S2: Table S1). Using the method described for *L. cardinalis*, we built a single raster layer representing the average *A. colubris* abundance during the flowering of each species and then extracted the abundance at the presence coordinates for each species. We then built a linear model with local *A. colubris* abundance as the response variable, *Lobelia* species as a factor predictor variable, and set *L. cardinalis* as the reference category so that coefficients indicate whether the econgeneric species differed from *L. cardinalis*.

For the second analysis, we explored the extent to which the week of local peak abundance of *A. colubris* predicts the date of flowering observations of *Lobelia* species, and whether the relationship between these two variables varies by species. For each *Lobelia* presence location, we determined the week (1–52) of the peak abundance of *A. colubris* during the year (eBirdst R package; Strimas‐Mackey et al., 2023). Presence locations with an average abundance of 0 (no peak) were removed from the data set (*N* = 21). Next, we used the numeric week of each presence observation (1–52) as a metric for the date of flowering observations. Here we assumed presences refer to flowering observations because we previously work by found the vast majority of *Lobelia* iNaturalist observations to be of plants in flower (Coffey and Simons, 2023). We then performed an offset Poisson regression model where the week of *Lobelia* observation acted as the response variable, *Lobelia* species acted as a factor predictor, and the week of peak *A. colubris* abundance (log transformed) acted as the offset term. We could thus effectively model the ratio of peak abundance week to *Lobelia* observation week. The model intercept was set to 0 such that coefficients indicate whether the ratio for each species significantly differs from 1, with ratios lower or higher than 1 indicating *Lobelia* observations before or after the week of peak abundance, respectively. Finally, to compare ratios between species, we performed pairwise comparisons with Tukey contrasts (R package emmeans; Lenth, 2023).

## RESULTS

### Model evaluation

**Figure 2:**
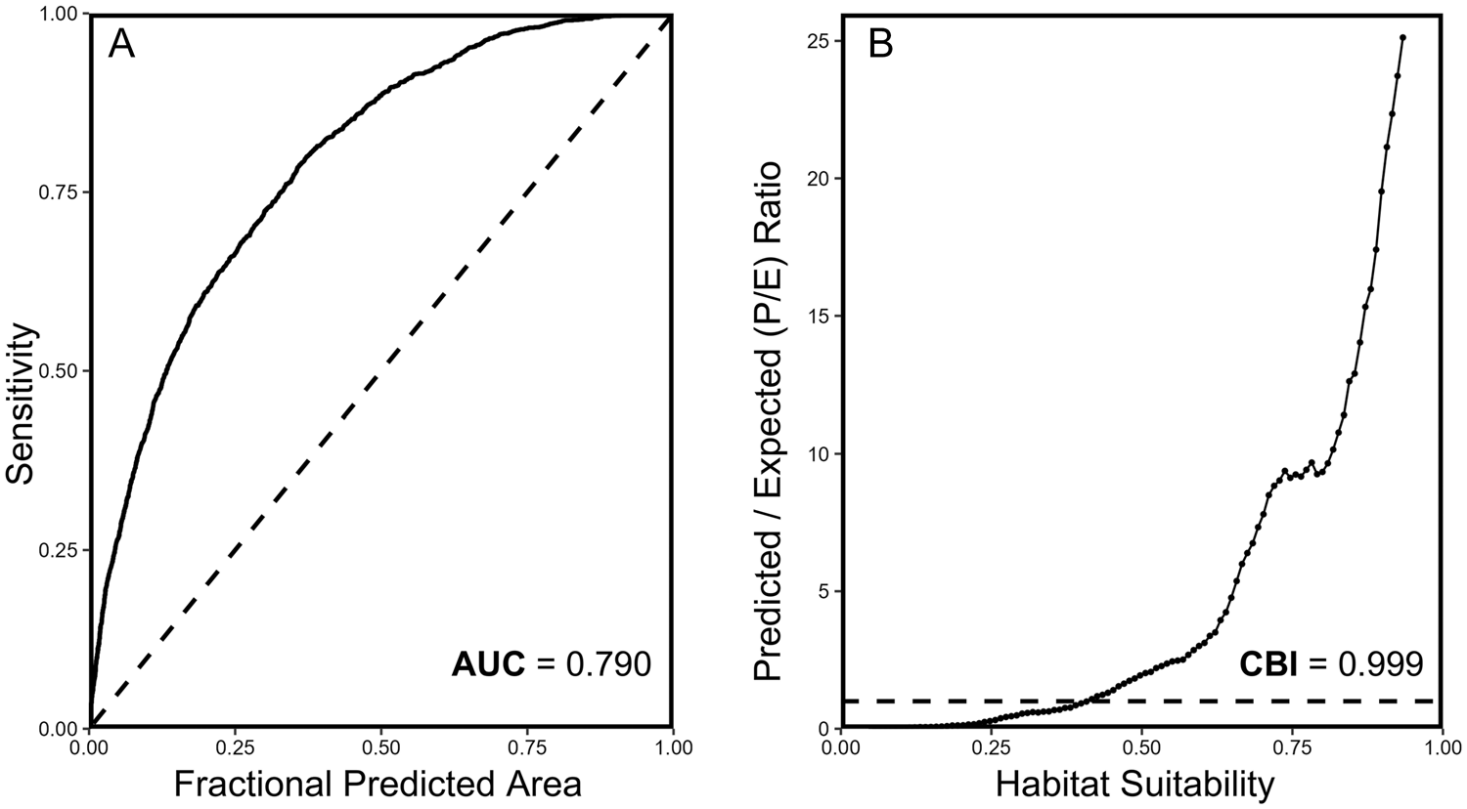
(A) Receiver operating curve for the final MaxEnt model. The dashed black line indicates the expected receiver operating curve of a random prediction. (B) Continuous Boyce index plot for the final MaxEnt model. The dashed black line indicates the curve of a completely random model (predicted/expected ratio = 1).

During cross validation, the AUC and CBI of the training data (80% of presences) were 0.791 and 0.998, respectively, and the AUC and CBI of the testing data (20% of presences) were 0.786 and 0.992, respectively. Because these values were above the AUC and CBI thresholds of 0.7 and 0.5, we built a final model using all presence records as training data. This final model had an AUC of 0.790 and a CBI of 0.999 (Figure 2). When the model was evaluated against the 2023 presence locations, the resulting AUC was 0.792 with a CBI of 0.999. The maxSSS cloglog threshold of the final model was 0.5061. As such, areas of the prediction map (Figure 1A) with habitat suitability values above 0.5061 were considered regions of likely presence of *L. cardinalis* (Figure 1B).

### Environmental variable contributions

#### Percent contribution

In the MaxEnt model, *A. colubris* abundance had a contribution value of 1.59% (Table 2), which ranked seventh among the environmental variables. The three variables with the highest relative contribution to model training were water cover, cropland cover, and tree cover (see Table 2). Adjusting the coefficients of these three variables was associated with ~76% of total increase in model gain during training.

#### Permutation importance

The abundance of *A. colubris* had a permutation importance of 0.37% (Table 2), which ranked 11th among the environmental variables. The three highest variables by permutation importance were cropland cover, water cover, and grassland cover—around 63% of the model's predictive capacity was explained by these three variables alone (Table 2).

#### Jackknife testing

**Figure 3:**
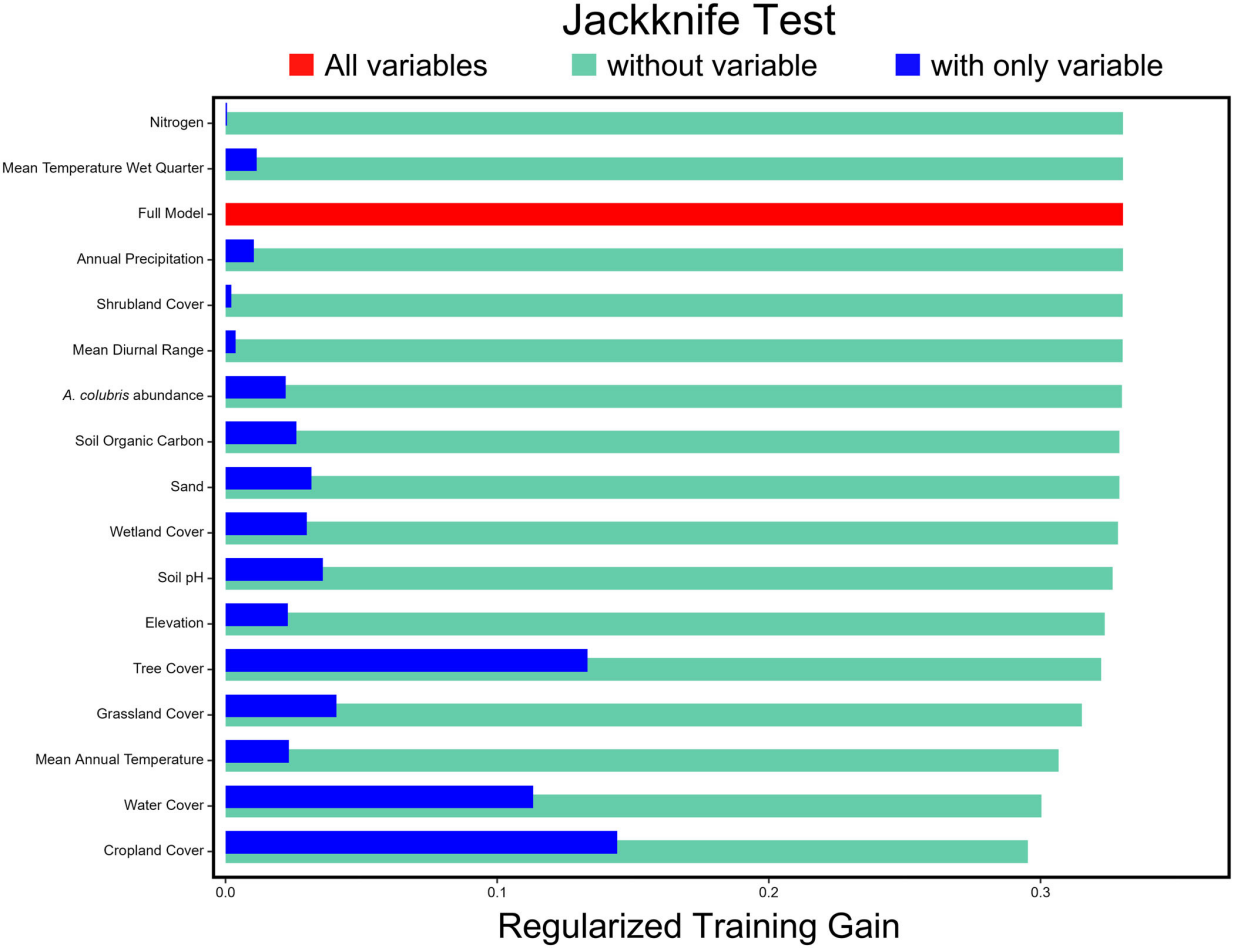
Bar plot showing the results of the jackknife tests. The red bar shows the regularized training gain of the full model, which includes all environmental variables. The turquoise bars show the training gain of models when a given variable was excluded from model building. The blue bars show the training gain of models where that particular variable was used alone to build the model. Variables are ordered from highest to lowest training gain when excluded from the model.

The regularized training gain of the full model (including all environmental variables) was 0.3304 (Figure 3). When *A. colubris* abundance was used alone to build the model, it resulted in a training gain of 0.0221, which ranked 11th among environmental variables. Comparatively, cropland cover (0.1442), tree cover (0.1333), and water cover (0.1132) had the highest training gains when used in isolation (Figure 3). The model training gain when *A. colubris* abundance was excluded from model building was 0.3300 (11th rank), indicating only a small loss in training gain relative to the full model (0.3304), whereas the exclusion of cropland cover from model building led to the lowest training gain (0.2954), followed by water cover (0.3003) and mean annual temperature (0.3067; Figure 3).

#### Variable response curves

**Figure 4:**
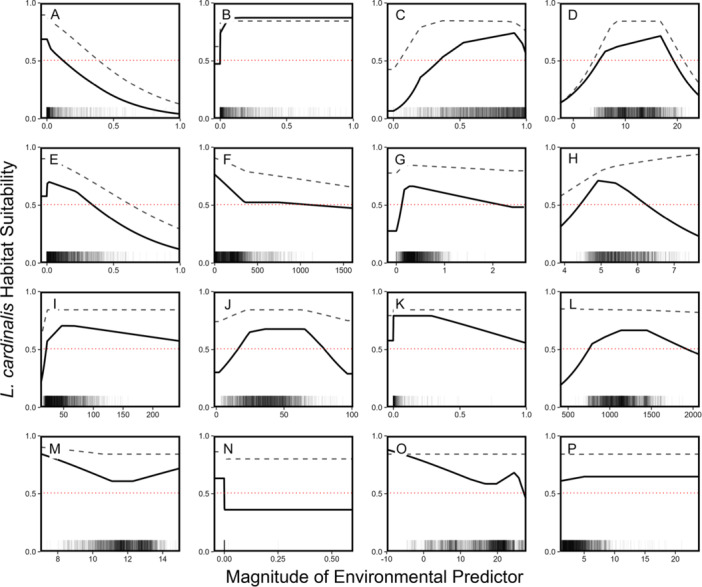
MaxEnt variable response curves (A–P) in descending order of the percent contribution of each variable to the model (see Table 1). Variables (with unit for each *x*‐axis in parentheses): (A) Cropland cover fraction (%), (B) water cover fraction (%), (C) tree cover fraction (%), (D) mean annual temperature (°C), (E) grassland cover fraction (%), (F) elevation (m a.s.l.), (G) *A. colubris* abundance, (H) soil pH, (I) soil organic carbon in fine earth (g·kg^−1^), (J) sand in fine earth (%), (K) wetland cover fraction (%), (L) annual precipitation (mm·m^−2^), (M) mean diurnal range (°C), (N) shrubland cover fraction (%), (O) mean temperature of the wettest quarter (°C), (P) total nitrogen (g·kg^−1^). Thick black lines show the response curves for models with variables built in isolation. Dashed grey lines show the marginal response curve (when all other environmental variables are held at their mean value). Rug plots along the *x*‐axis show the values of the environmental predictor variables at the 1747 presence locations for *L. cardinalis*. The horizontal dotted red line indicates the maxSSS presence–absence threshold (0.5061).

Figure 4 presents the variable‐in‐isolation curve and the marginal response curve for the 16 environmental variables with panels ordered (A–P) from highest to lowest percent contribution (Table 2). Regions where the curves rise above maxSSS cloglog threshold, indicated by the dotted red line, show the range of environmental values associated with likely presence of *L. cardinalis*. When used in isolation, *A. colubris* abundance showed a plateaued bell‐shaped response curve, with habitat suitability being low in the absence of *A. colubris*, quickly rising to a peak around an abundance of ~0.3, and gradually declining in habitat suitability toward regions of the highest abundance (Figure 4G; thick black line). The marginal response curve was much flatter and demonstrated higher habitat suitability across the board (Figure 4G; thin grey dashed line). In the variable‐in‐isolation response curve, *L. cardinalis* presence, indicated by habitat suitability above the maxSSS threshold of 0.5061 (dotted red line), occurred in regions with *A. colubris* abundance between 0.12 and 2.14 (Figure 4G). On the other hand, the entire marginal response curve fell above the maxSSS presence threshold (Figure 4G).

### Species comparisons

**Figure 5:**
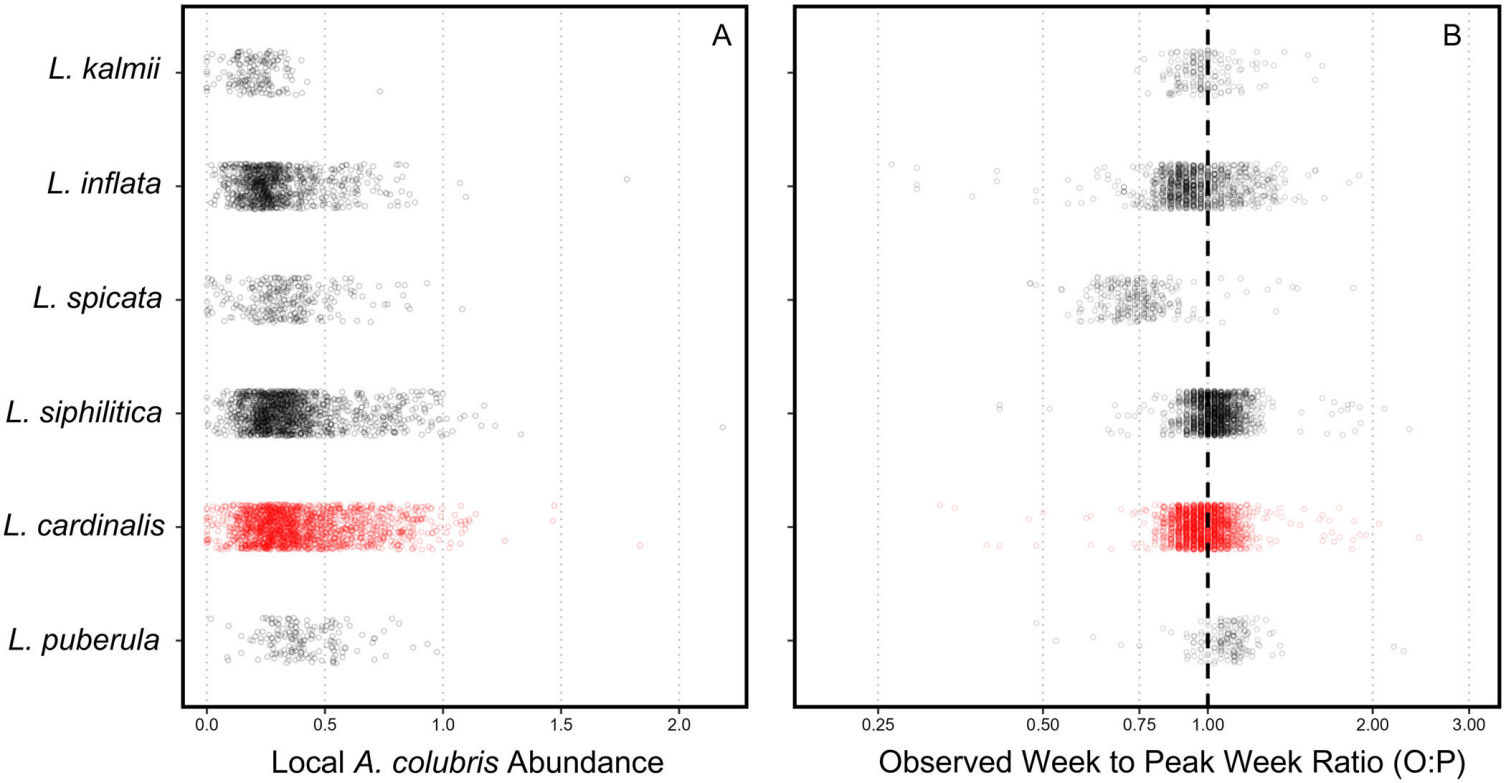
(A) Local *Archilochus colubris* abundance at presence locations of six *Lobelia* species native to eastern North America during their flowering period (see Appendix S5). Species are ordered from lowest (top) to highest (bottom) average local abundance of *A. colubris*. (B) The ratio of the week of observation of each *Lobelia* presence location with respect to the week of peak *A. colubris* abundance at the presence location. The *x*‐axis has been log‐transformed. The dashed black vertical line indicates a ratio value of 1, which signifies a *Lobelia* presence being observed on the week of peak *A. colubris* abundance. Points that fall to the left of the dashed line indicate presence locations where the *Lobelia* was observed before peak abundance; points to the right of the dashed line indicate presence locations where the *Lobelia* was observed after peak abundance.

All *Lobelia* species, except for *L. puberula*, had significantly lower local abundance of *A. colubris* during their flowering season when compared to the reference category, *L. cardinalis* (Figure 5A). Presence locations of *L. cardinalis* had a predicted *A. colubris* abundance value of 0.396 (SE = 0.0047; Figure 5A, Appendix S2: Table S3).

All *Lobelia* species except for *L. kalmii* had a ratio of observation week to peak abundance week that significantly differed from 1 (Figure 5B; Appendix S2: Table S4). *Lobelia cardinalis* had a predicted ratio of 0.985 (95% CI 0.977–0.993; Figure 5B). *Lobelia spicata*, the earliest‐flowering species in the analysis (Appendix S2: Table S1) had a predicted ratio of 0.749 (95% CI 0.731, 0.766), whereas *L. puberula*, the latest‐flowering species (Appendix S2: Table S1) had a predicted ratio of 1.079 (95% CI 1.052, 1.106; Figure 5B). Tukey contrasts indicated that the week‐to‐week ratio for *L. cardinalis* was significantly different from all species except *L. kalmii* (Appendix S2: Table S5).

## DISCUSSION

Plant–pollinator mutualisms have the potential to influence plant species distributions (Chalcoff et al., 2012; Wisz et al., 2013). Eastern North American populations of *L. cardinalis*, a species known to be obligately outcrossing and suffer high rates of inbreeding depression, have long been thought to be solely pollinated by *A. colubris*, the only hummingbird species in the region (Bertin, 1982; Devlin and Stephenson, 1984, 1985; Johnston, 1992; Bartkowska and Johnston, 2014). If access to *A. colubris* is critical for successful reproduction in *L. cardinalis*, the plant's distribution should be controlled by the occurrence and/or abundance of *A. colubris*. In the context of a species distribution model, we predicted that local abundance of *A. colubris* will be an important factor in limiting the distribution of *L. cardinalis*, relative to other potential environmental factors and that habitat suitability will be highest in regions of the highest *A. colubris* abundance. Moreover, given that congeneric eastern North American *Lobelia* species are not hummingbird‐pollinated, we predicted that local abundance of *A. colubris* would be higher in regions where *L. cardinalis* occurs when compared to congenerics and that the flowering period of *L. cardinalis* in these regions would strongly overlap with the point of peak local hummingbird abundance.

Our first prediction that the magnitude of local *A. colubris* abundance should be a key variable in the MaxEnt distribution model of *L. cardinalis* did not appear to be well supported by our results. Among the set of 16 environmental variables, *A. colubris* abundance was consistently ranked as a mid‐ to low‐contributing variable across all three variable contribution metrics with several other predictors consistently suggested as more important drivers of the distribution of *L. cardinalis* (Table 2, Figure 3). In addition, the flat marginal response curve for hummingbird abundance, which fell entirely above the maxSSS threshold, indicated that when controlling for these other environmental factors, hummingbird abundance had little effect on the final prediction of the distribution of *L. cardinalis*. In the absence of objective expectations for the contribution of biotic factors relative to abiotic factors that are typically included in models, it would be inappropriate to conclude that *A. colubris* abundance is unimportant. Clearly, further application of MaxEnt models that include biotic predictors from known specialist relationships is needed to resolve this issue. Likewise, we found no strong support for our hypothesis through our second prediction, that habitat suitability for *L. cardinalis* would be highest in regions of the highest *A. colubris* abundance. We found that in the absence of *A. colubris*, habitat suitability was indeed low, but once *A. colubris* abundance reached a particular level (i.e., ~0.3), habitat suitability plateaued, even gradually decreasing toward regions with the highest abundance (Figure 4G). However, our predictions regarding differences among congeneric *Lobelia* species provided clear support for our hypothesis. The abundance of *A. colubris* at presence locations of *L. cardinalis* during flowering was indeed higher than all but one of the analyzed congenerics, and flowering of *L. cardinalis* was typically observed near the week of peak *A. colubris* abundance, suggesting that *L. cardinalis* tends to flower when *A. colubris* is most prevalent in the local environment.

**Figure 6:**
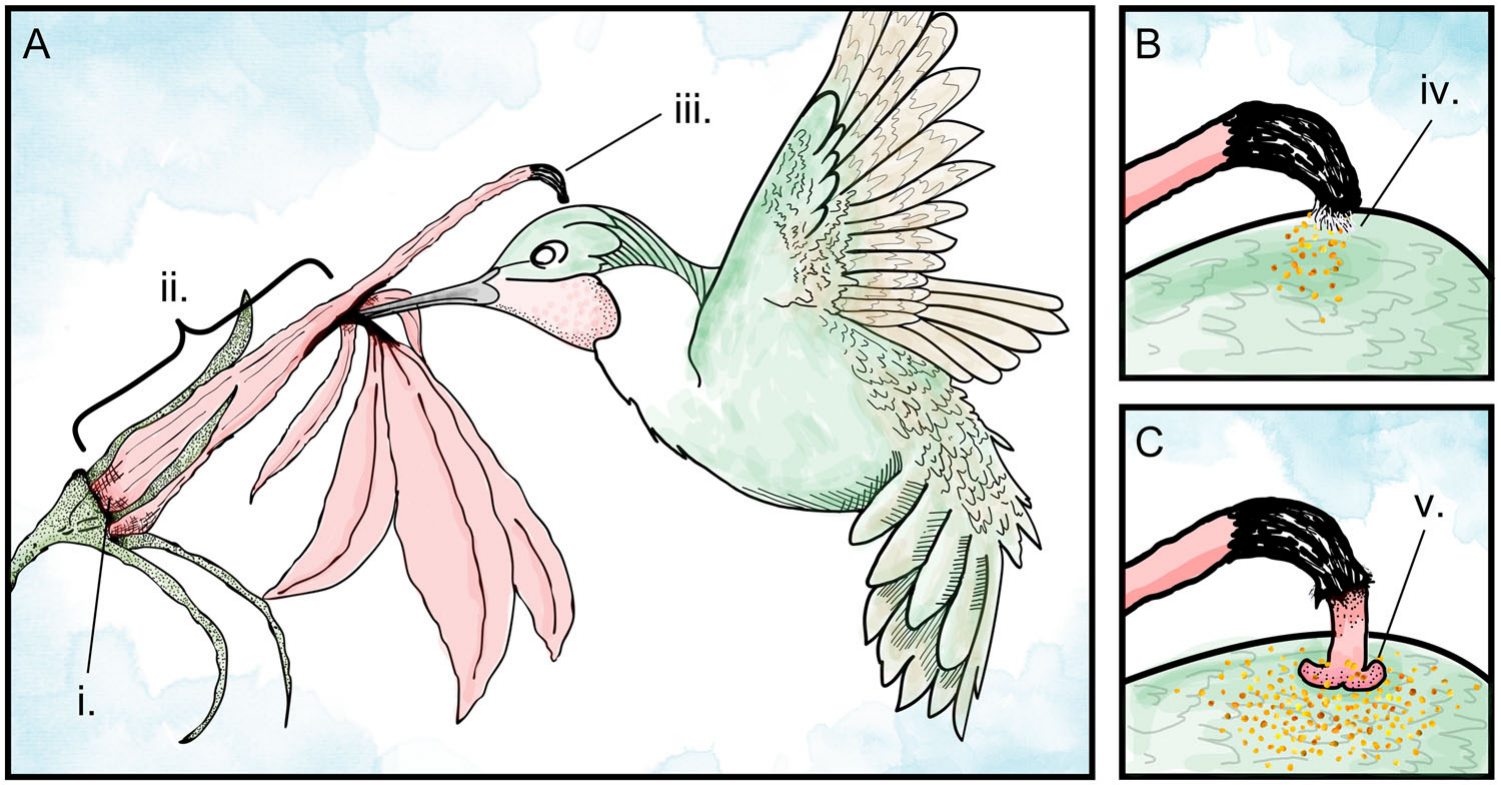
(A) Illustration of a Ruby‐Throated Hummingbird (*Archilochus colubris*) visiting a cardinal flower (*Lobelia cardinalis*). (i) Point of nectar access inside the corolla tube at the junction between the base of the stamen tube and the ovary. (ii) Length of the corolla tube. (iii) Tip of the stamen tube (fused anthers), which contacts the body of a visiting hummingbird. (B) Close‐up of staminate phase of *L. cardinalis* flower contacting a visiting hummingbird. (iv) Contact with the brush‐like hairs on the ventral side of the anther tube instigates the release of pollen from the anther tube. (C) Close‐up of pistillate phase of *L. cardinalis* flower contacting a visiting hummingbird. (v) Receptive stigma lobes contacting hummingbird body and any carried pollen.

The simplest explanation for *A. colubris* abundance not being a key variable in the distribution model is that *A. colubris* is not truly the sole pollinator of *L. cardinalis*. A previous phenetic analysis of *L. cardinalis* herbarium specimens revealed a maximum spatial extent far beyond the traditional summer range of *A. colubris*, implying other hummingbird species likely act as pollinators outside our study region (Thompson and Lammers, 1997). In the southwestern‐most extent of our study region (Texas and Oklahoma), there are populations of Black‐Chinned Hummingbirds (*Archilochus alexandri*), which likely visit and pollinate *L. cardinalis* (Fink et al., 2023). However, the presence of an additional hummingbird species in a small region is unlikely to explain the lower‐than‐expected importance of *A. colubris* abundance in our model. Still, within the eastern North American range of *L. cardinalis*, the importance of potential non‐hummingbird pollinators remains to be determined. Pollen removal and deposition by *A. colubris* during visitation is associated with nectar access; flowers are structured such that visiting hummingbirds are optimally positioned to contact the stamen tube to both receive and deposit pollen (Figure 6; Devlin and Stephenson, 1984, 1985; Devlin et al., 1987). It is unknown whether insects, which are known anecdotally to visit *L. cardinalis* (M. Coffey, personal observations), are large enough to trigger the pollen deposition mechanism upon a floral visit. Pollen may also be released onto the lower three petals as flowers transition from staminate to pistillate phase (M. Coffey, personal observations), providing another potential mechanism for insect visitors to obtain pollen, but how often insects might receive pollen in this way is unknown. Therefore, further field study is required to understand the frequency that *L. cardinalis* flowers are visited by insects and whether these visitors can act as functional pollinators.

Another explanation for the weak contribution of *A. colubris* abundance to the distribution model is associated with a key caveat of our model framework: the chosen spatial extent. We decided upon –100° longitude as an arbitrary boundary line for eastern North America. The range boundary of *A. colubris* is slightly east of this line (Appendix S1: Figure S3); however, the random sampling of pseudo‐absence points was sparse near this western boundary (Appendix S1: Figure S1). It is possible that were we to use the entirety of North America as our boundary, the importance of *A. colubris* abundance to model building may have risen, given that more background points would have been sampled from regions in which *A. colubris* was absent. For example, Huang et al. (2021) found that variable contributions to MaxEnt models can vary depending on the scale of the spatial extent. To explore the potential impact of spatial extent, we performed a supplementary analysis where we expanded our model's spatial extent to include all the United States and Canada (western boundary of –130° longitude). We found that this change drastically increased the variable importance metrics for *A. colubris* abundance, but the model fit an identically shaped response curve (Appendix S1: Figures S5 and S6). Such a result could indicate that the simple presence, rather than the abundance, of hummingbirds may be important for *L. cardinalis* distribution; within the range of *A. colubris*, fine scale variation in abundance is not critical for determining optimal environments for *L. cardinalis*.

Hummingbirds are often considered more effective pollinators than insects; compared to bees, hummingbirds fly longer distances thereby interacting with more plant individuals, have larger ranges (i.e., >1 km), groom less, do not feed on pollen, and are more efficient at pollen removal, pollen deposition, and pollen transfer (Castellanos et al., 2003; Krauss et al., 2017; Mackin et al., 2021; Abrahamczyk, 2019, 2022). It is possible that low numbers of hummingbirds (or even locally a single bird) could function as effective pollinators for a cardinal flower population. For example, the 19th‐century botanist Thomas Meehan (1902) once noted that fertility did not differ between garden populations of *L. cardinalis* and *L. siphilitica* (~50 individuals each), despite large differences in pollinator visitation rates—having only witnessed a single instance of an *A. colubris* individual visiting his Cardinal Flower population. The potential efficacy of *A. colubris* pollinators at low numbers may also explain why models at both spatial extents predicted declines in habitat suitability in regions of the highest *A. colubris* abundance (Figure 4G; Appendix S1: Figure S5). Although a region might have high *A. colubris* abundance, hummingbirds are also likely visiting co‐flowering plant species, leading to potential competition for visitation (Jones et al., 2012; Johnson et al., 2022). Therefore, if *A. colubris* is an effective pollinator at low numbers, it is reasonable that optimal *L. cardinalis* habitat suitability might occur in regions with mid to low levels of *A. colubris* abundance to avoid such competition.

A final explanation for the less‐than‐expected importance of *A. colubris* abundance is the potential for evolution toward higher rates of self‐fertilization in regions where *A. colubris* is in low abundance or absent. Greater self‐compatibility and higher rates of self‐fertilization are known to occur near range or environmental margins (Cwynar and MacDonald, 1987; Abbott and Gomes, 1989; Ledig et al., 2005; Griffin and Willi, 2014) and are typically hypothesized to be favored as a mechanism of reproductive assurance (Barrett and Harder, 2017). Geographic variation in mating system traits has been noted several times in the *Lobelia* genus, with spatial variation of gynodioecious floral morphs in *L. siphilitica* and *L. spicata* (Caruso and Case, 2007; Miller and Stanton‐Geddes, 2007; Byers, 2020) and latitudinal variation in style exsertion in *L. inflata* (Hughes and Simons, 2015; Coffey and Simons, 2023). Though *L. cardinalis* is known to suffer high inbreeding depression, it is also self‐compatible (Johnston, 1992). It is possible that *L. cardinalis* populations occurring in regions with low or absent *A. colubris* may have evolved toward greater selfing, reducing their reliance on external pollination. However, given that *L. cardinalis* flowers express protandry, any increase in self‐fertilization would either require shifts toward staminate and pistillate phase overlap or increased geitonogamy. Cardinal flower individuals demonstrate Darwin's inflorescence syndrome, where staminate phase flowers are borne above pistillate phase flowers, which is thought to reduce geitonogamy by exploiting the typical upward foraging behaviour of pollinators; pollinators deposit pollen on pistillate flowers first before collecting pollen from staminate flowers (Devlin and Stephenson, 1985; Strelin et al., 2024). Flower visitation of *L. cardinalis* by *A. colubris* typically occurs in an acropetal manner, where birds begin foraging in the middle of the raceme and then proceed upward (Devlin and Stephenson, 1985). Studies of marginal *L. cardinalis* populations could clarify whether there is increased overlap of a flower's sexual phases or changes in hummingbird foraging behavior.

Rather than *A. colubris* abundance, several environmental variables were found to be the most likely drivers of the distribution of *L. cardinalis* across eastern North America: water cover, cropland cover, tree cover, grassland cover, and mean annual temperature. The habitat of *L. cardinalis* has traditionally been defined by shaded water edges (lakes, rivers, streams; McVaugh, 1936, 1940; Bowden, 1959). Thus, the influence of water cover and tree cover to the distribution model is not surprising. The variable response curves are also consistent with expectations given these traditional habitat features: habitat suitability was highest when water cover was greater than 0% and at high tree cover fractions (Figures 4B and 4C, respectively). Such regions would be categorized by highly wooded areas with water bodies, providing optimal conditions for shaded, water‐edge habitats. Alternatively, cropland and grassland cover, both characterized by strong negatively trending response curves, indicate that *L. cardinalis* populations avoid agricultural lands and open grasslands (Figures 4A and 4E). Agricultural land, dominated by crops or livestock grazing, is likely inhospitable habitat space for wild plant species and, unlike congeners, *L. cardinalis* is not typically known to grow in disturbed sites, which may be more common in regions with abundant agricultural land, or in open fields and grasslands (Bowden, 1959; Baskin and Baskin, 1992; Pigliucci et al., 1997; Byers, 2020).The importance of mean annual temperature is unsurprising given that temperature is commonly a key variable in MaxEnt models (Bradie and Leung, 2017). The bell‐shaped response curve fit to mean annual temperature likely indicates the thermal limits for *L. cardinalis* (Figure 4D), although the cold limit is probably more accurate than the warm, given that the warmest area in the study region, Florida, does not represent the southernmost range limit of the species (Thompson and Lammers, 1997). Finally, it is important to note that the AUC of our final model was 0.790 (Figure 2)—which indicates fair model quality—yet this value is not as high as those reported in some other MaxEnt modelling studies. This lower value could suggest that that some of the remaining variation in the distribution of *L. cardinalis* may be explained by environmental factors not included in the model.

One final caveat is our choice of a 1‐km spatial scale (grain size) for our model. We used a 1‐km grain size because it is a common spatial scale used in MaxEnt modelling and the native scale for the spatial data sets in the R package geodata (Hijmans et al., 2023a). Using 1‐km grain size could influence the importance of a variable if its variation at a fine scale is lost when it is aggregated to a larger grain size. For example, in our analysis, we used four SoilGrids soil variables (Poggio et al., 2021), which each had low importance across variable contribution metrics. Given the spatial scale of the analysis, these results could be misleading if the soil variables are unreliable metrics of true soil conditions at a 1‐km grain size. Miller, Blackwood and Case (2024) found that when comparing measured values for field soil samples to predicted values from SoilGrids models at a 250‐m grain size, the predictions for soil texture variables were generally reliable, but predictions for soil carbon and nitrogen were not. Therefore, the importance of the soil variables in our model may not reflect their true importance to the distribution of *L. cardinalis*; thus, it is difficult to make claims that other variables might be more important than soil features. The choice of grain size may also explain the high importance of the land‐cover variables to the model. Land‐cover variables are the most granular; they are often negatively spatially autocorrelated, with adjacent raster cells strongly differing from one another. The greater between‐cell variation of the land‐cover variables at this grain size may explain why they were important to the model across contribution metrics—they were critical in differentiating presence points from background points. However, the importance of the landcover variables to the model is not spurious: Were the grain size smaller (i.e., 250 m or 100 m), a similar strong negative spatial autocorrelation would likely still be shown, and thus we expect their importance would remain.

## CONCLUSIONS

Using presence data from iNaturalist community science observations, we found no strong evidence that the spatial distribution of *L. cardinalis* is predominantly driven by the local abundance of its specialist pollinator, *A. colubris*. However, we also show that when compared to congenerics, *L. cardinalis* occurs in regions with higher abundance and tends to flower proximal to the point of peak local hummingbird abundance. These findings suggest that even though *L. cardinalis* generally occurs in regions with greater abundance of *A. colubris* and flowers when hummingbirds are most active, fine‐scale variation in the local magnitude of *A. colubris* abundance does not appear to drive the spatial distribution of *L. cardinalis*.

This work provides insight into how plant–pollinator mutualisms can influence plant species distributions. However, that the magnitude of *A. colubris* abundance was not found to be a core driver of the spatial distribution of *L. cardinalis* is puzzling. Therefore, further study of *L. cardinalis*—specifically flower visitation and marginal population dynamics—is required. Finally, this work demonstrates how traditional species distribution modelling tools like MaxEnt, which are typically used in conservation biology, can be applied to study the relationship between pollinators and plant spatial distributions.

## AUTHOR CONTRIBUTIONS

M.L.C: conceptualization, methodology, data curation, analysis, visualization, and writing; A.M.S. conceptualization, methodology, funding acquisition, supervision, and writing.

## Supporting information


**Appendix S1.** Supplementary figures.
**Figure S1.** Map of distribution of *Lobelia cardinalis* presence locations (*N* = 1747; red points) and the random background points (*N* = 10,000; black points).
**Figure S2.** Histogram showing the distribution of iNaturalist observation dates for (A) *Lobelia spicata*, (B) *L. inflata*, (C) *L. kalmii*, (D) *L. cardinalis*, (E) *L. siphilitica*, and (F) *L. puberula*.
**Figure S3.** Average *Archilochus colubris* abundance (1 July to 13 October) across eastern North America.
**Figure S4.** Cluster dendrogram showing the correlations between environmental predictor variables based on pairwise Pearson correlation coefficients between raster layers (10% sample of all raster cells).
**Figure S5.** Variable response curves for *Archilochus colubris* abundance at (A) the spatial extent of eastern North America (main model) and (B) the spatial extent of all North America (supplementary model).
**Figure S6.** (A) Continuous habitat suitability map prediction for the supplementary MaxEnt model built at the spatial extent of all North America.
**Figure S7.** Bar plot showing the results of the jackknife tests for the supplementary MaxEnt model that used *Lobelia cardinalis* presence data from 2018 to 2022.
**Figure S8.** (A) The marginal and (B) variable‐in‐isolation response curves for the supplementary MaxEnt model that used *Lobelia cardinalis* presence data from 2018 to2022.

**Appendix S2.** Supplementary tables.
**Table S1.** *Lobelia* species used in the species comparison analyses.
**Table S2.** The results of the MaxEnt model optimization procedure performed with the R package ENMeval.
**Table S3.** Linear model summary for the species comparison analysis focusing on between‐species variation in local flowering season abundance of *Archilochus colubris*.
**Table S4.** Generalized linear model summary for the species comparison analysis focusing on between‐species variation in the ratio of *Lobelia* observation week to peak week of abundance of *Archilochus colubris*.
**Table S5.** Model summary table of the Tukey contrasts from the species comparison analysis focusing on between‐species variation in the ratio of *Lobelia* observation week to peak week of abundance of *Archilochus colubris*.
**Table S6.** Model evaluation metrics for the supplementary MaxEnt model, which used *Lobelia cardinalis* presence data from 2018 to 2022.
**Table S7.** Percent contribution (PC) and permutation importance (PI) of each environmental variable for the supplementary MaxEnt model, which used *Lobelia cardinalis* presence data from 2018 to 2022.

## Data Availability

The data that support the findings of this study are openly available through GBIF and are also available through the Figshare Digital Repository at https://figshare.com/s/5616a35dffd7eab2203f. The code used for the analysis and data visualization is also openly available through the Figshare Digital Repository at https://figshare.com/s/fd40c2840efdfeba3c75.
