# Supplementary material for: The spatial distribution of a hummingbird‐pollinated plant is not strongly influenced by hummingbird abundance
Source: Am J Bot. 2025 Apr 26;112(5):e70034. doi: 10.1002/ajb2.70034 (PMC12094066; doi:10.1002/ajb2.70034)
Supplement: Supplementary file 1 — Appendix S1. Supplementary figures. Figure S1. Map of distribution of Lobelia cardinalis presence locations (N = 1747; red points) and the random background points (N = 10,000; black points). Figure S2. Histogram showing the distribution of iNaturalist observation dates for (A) Lobelia spicata, (B) L. inflata, (C) L. kalmii, (D) L. cardinalis, (E) L. siphilitica, and (F) L. puberula. Figure S3. Average Archilochus colubris abundance (1 July to 13 October) across eastern North America. Figure S4. Cluster dendrogram showing the correlations between environmental predictor variables based on pairwise Pearson correlation coefficients between raster layers (10% sample of all raster cells). Figure S5. Variable response curves for Archilochus colubris abundance at (A) the spatial extent of eastern North America (main model) and (B) the spatial extent of all North America (supplementary model). Figure S6. (A) Continuous habitat suitability map prediction for the supplementary MaxEnt model built at the spatial extent of all North America. Figure S7. Bar plot showing the results of the jackknife tests for the supplementary MaxEnt model that used Lobelia cardinalis presence data from 2018 to 2022. Figure S8. (A) The marginal and (B) variable‐in‐isolation response curves for the supplementary MaxEnt model that used Lobelia cardinalis presence data from 2018 to2022. [file AJB2-112-e70034-s002.docx]

**Appendix S2.** Supplementary figures.


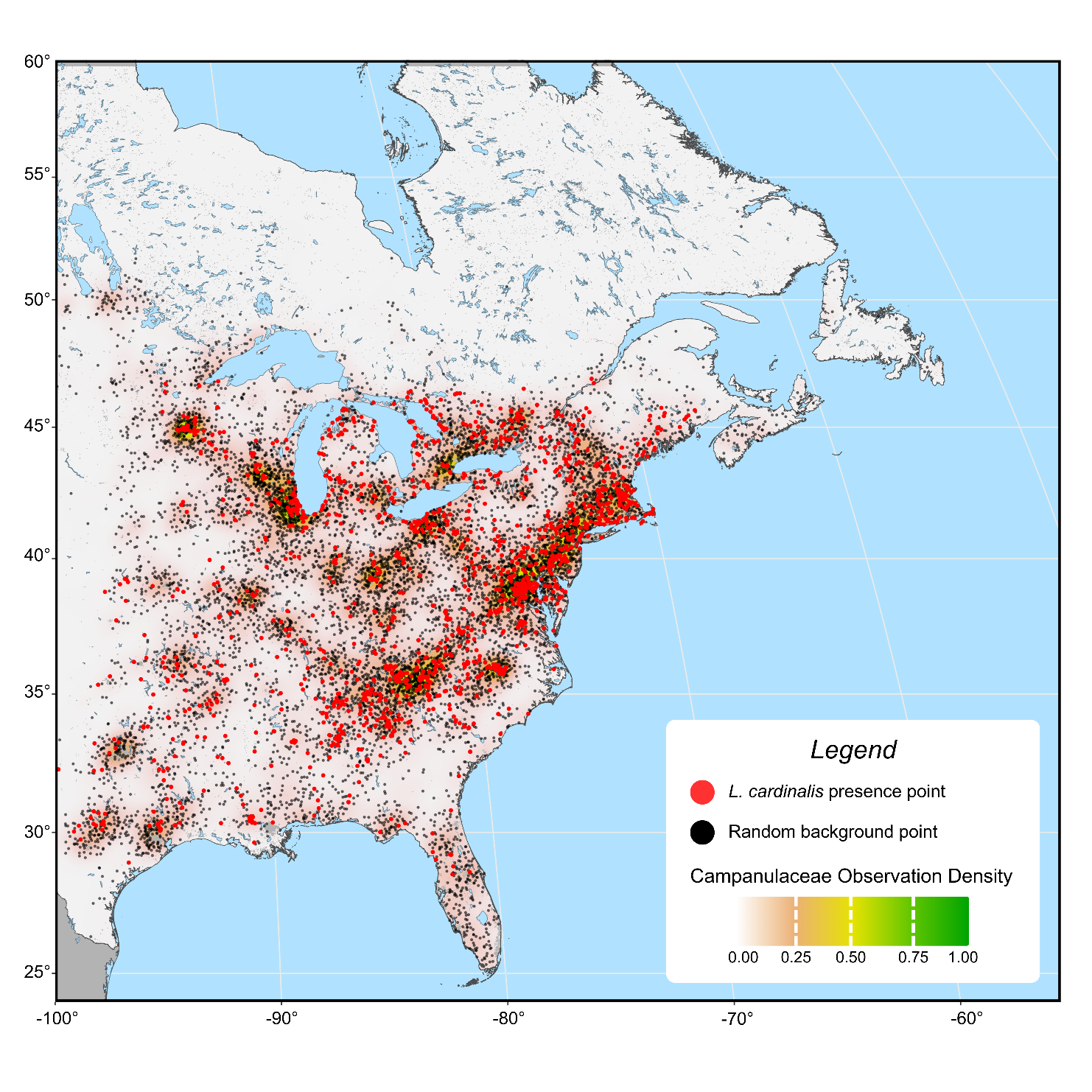


**Figure S1.** Map of distribution of Lobelia cardinalis presence locations (N = 1747; red points) and the random background points (N = 10,000; black points). Points are overlayed onto the 2D kernel density estimate of Campanulaceae observations that was used for the target-group bias correction of the random background points. Regions outside the model study area are in grey. Map is displayed using a Mollweide equal-area projection with a central meridian at –100º longitude.


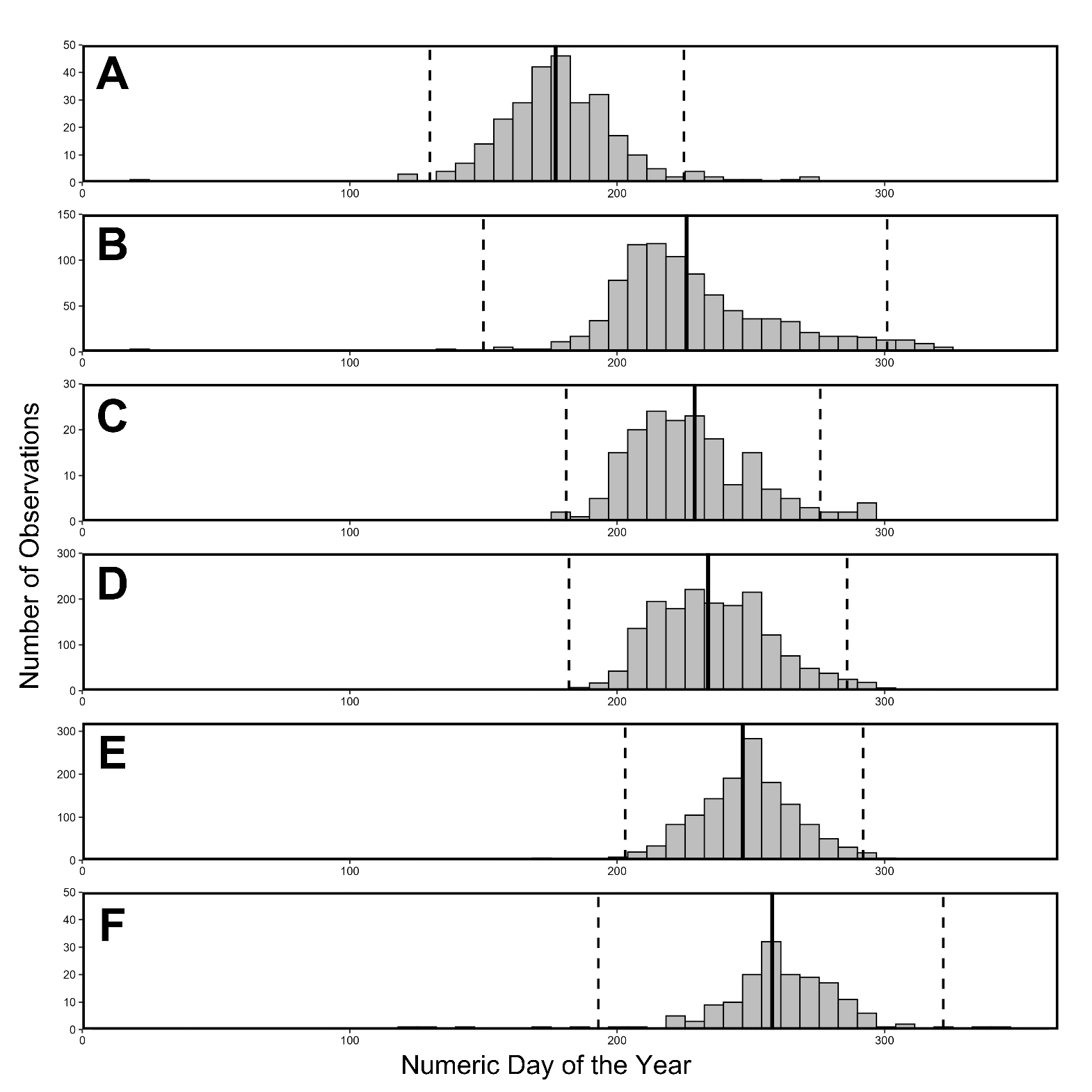


**Figure S2.** Histogram showing the distribution of iNaturalist observation dates for (A) Lobelia spicata, (B) L. inflata, (C) L. kalmii, (D) L. cardinalis, (E) L. siphilitica, and (F) L. puberula. Each bin shows the weekly count of observations (52 bins). The thick black vertical lines indicate the mean observation date of the species. The thin dashed vertical lines indicate 2SD above and below the mean observation date, used as the estimate of each species’ flowering period in the analyses. Plots are ordered (A–F) from earliest to latest mean observation date.


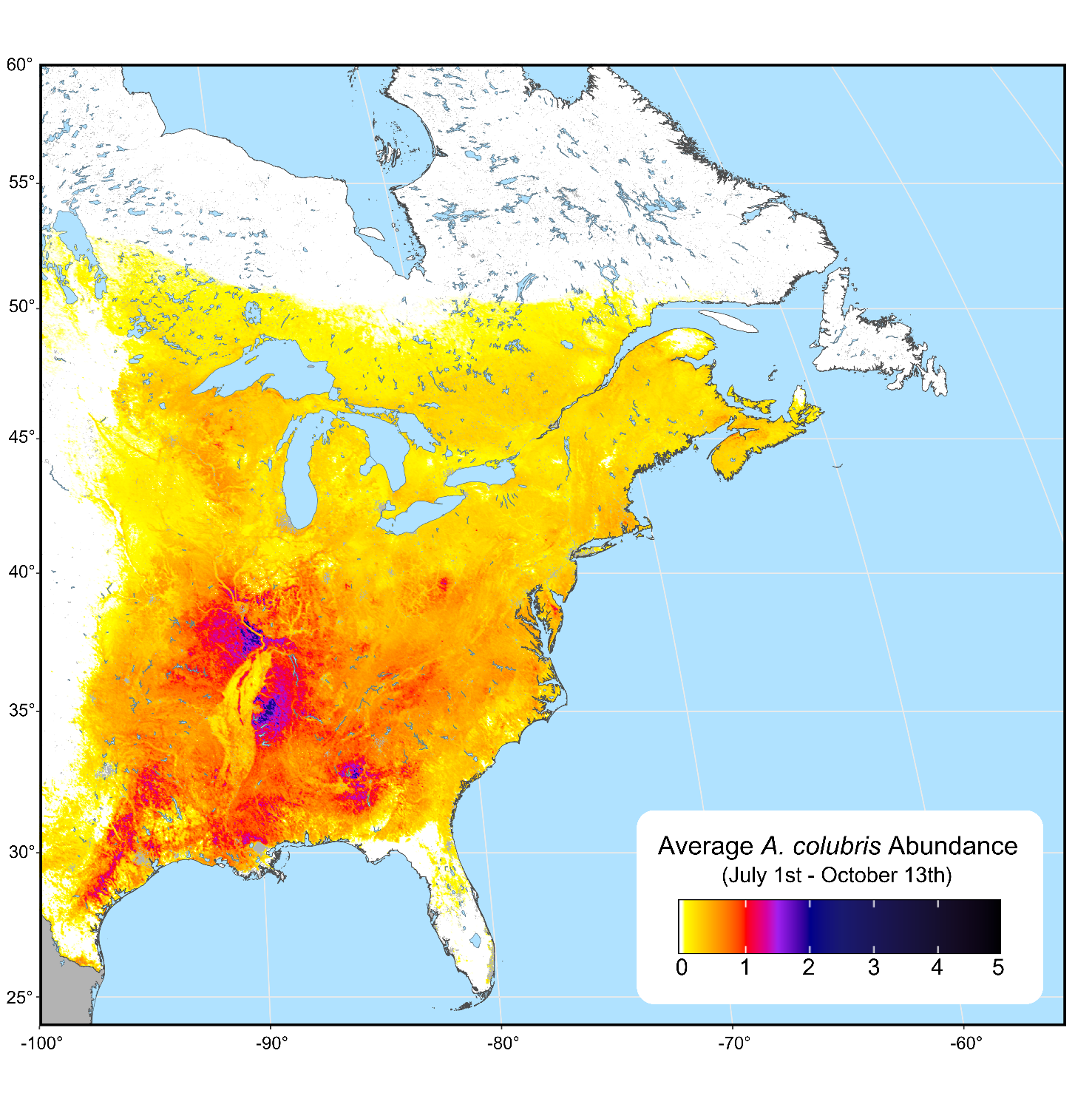


**Figure S3.** Average Archilochus colubris abundance (1 July–13 October) across eastern North America. Scale colors are formatted such that regions of the map shown in white denote areas with an average abundance of 0, indicating absence of A. colubris. Regions outside the model study area are in grey. Map is displayed using a Mollweide equal-area projection with a central meridian at –100º longitude.


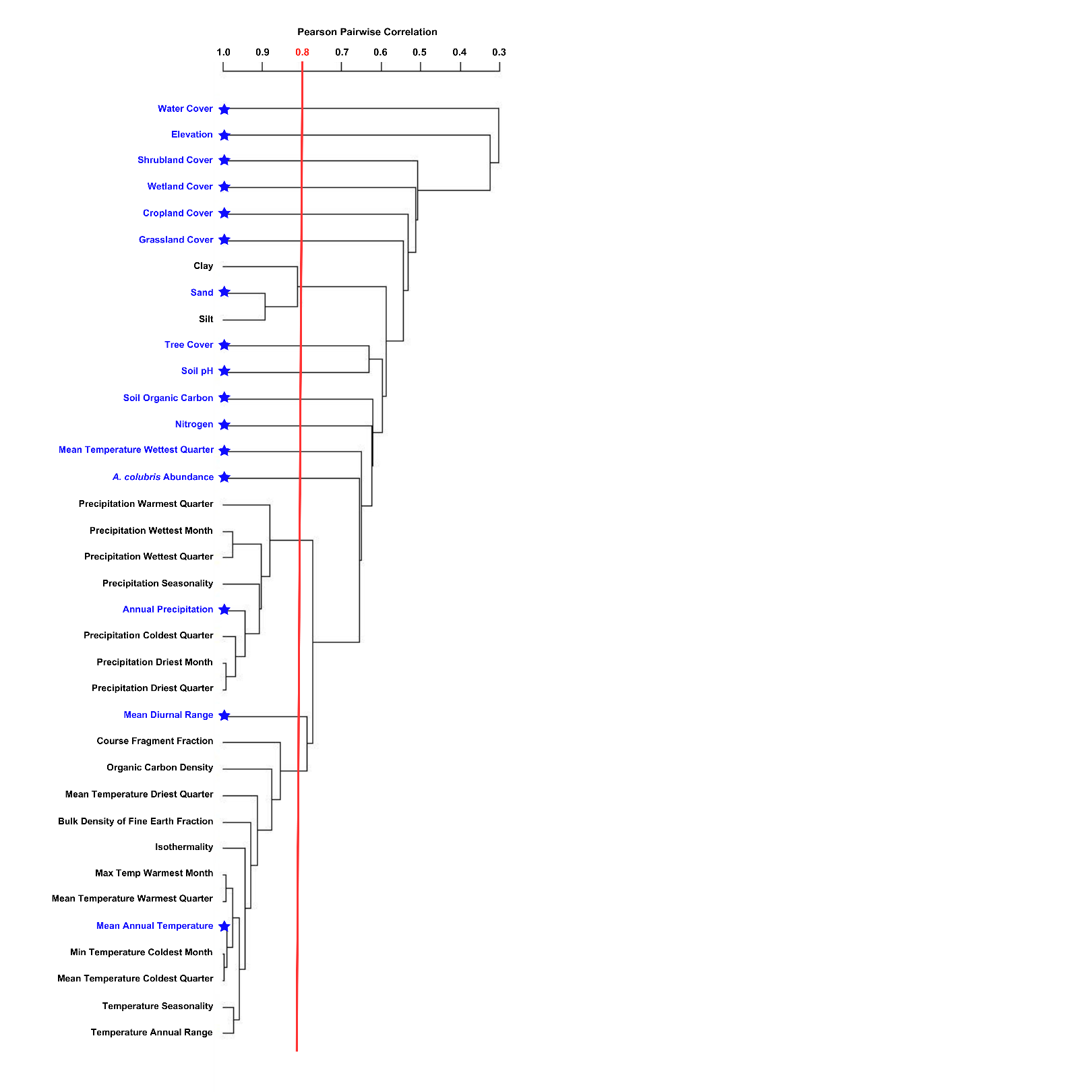


**Figure S4.** Cluster dendrogram showing the correlations between environmental predictor variables based on pairwise Pearson correlation coefficients between raster layers (10% sample of all raster cells). Nodes to left of the red line indicate clusters of environmental variables with |r| > 0.8. Blue text and stars indicate variables selected to be used in MaxEnt modelling.


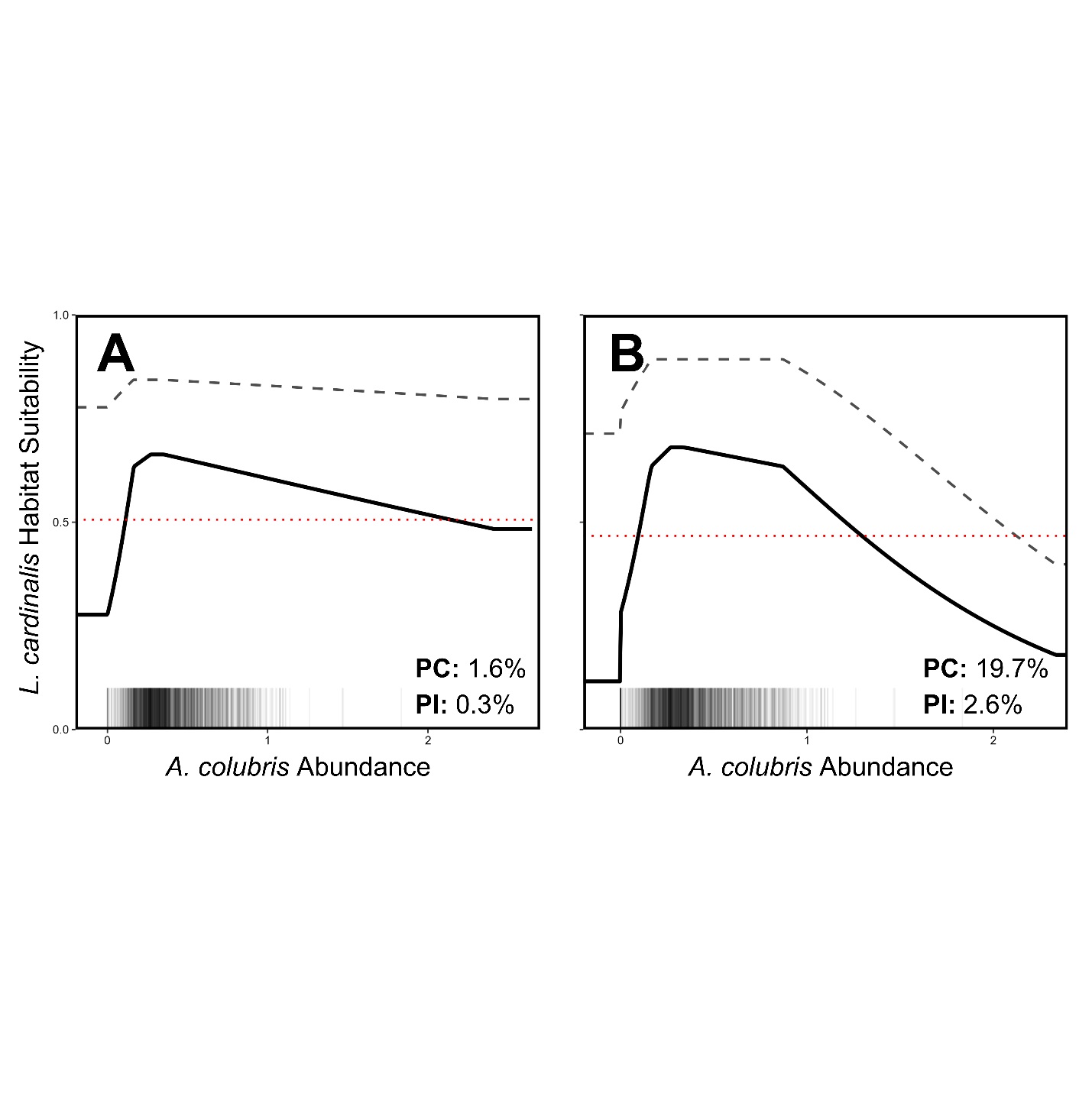


**Figure S5.** Variable response curves for Archilochus colubris abundance at (A) the spatial extent of eastern North America (main model) and (B) the spatial extent of all North America (supplementary model). The percent contribution (PC) and permutation importance (PI) of the A. colubris abundance variable model are presented in the bottom right corner of each plot. Thick black lines show the response curves for the models built with only A. colubris abundance (variable-in-isolation). Dashed grey lines show the marginal response curves (when all other environmental variables are held at their mean value). Rug plots along the x-axis show the raw A. colubris abundance values at the Lobelia cardinalis presence locations used to build the model. The horizontal dotted red line indicates the maxSSS presence/absence threshold of each model (A = 0.5061, B = 0.4671).


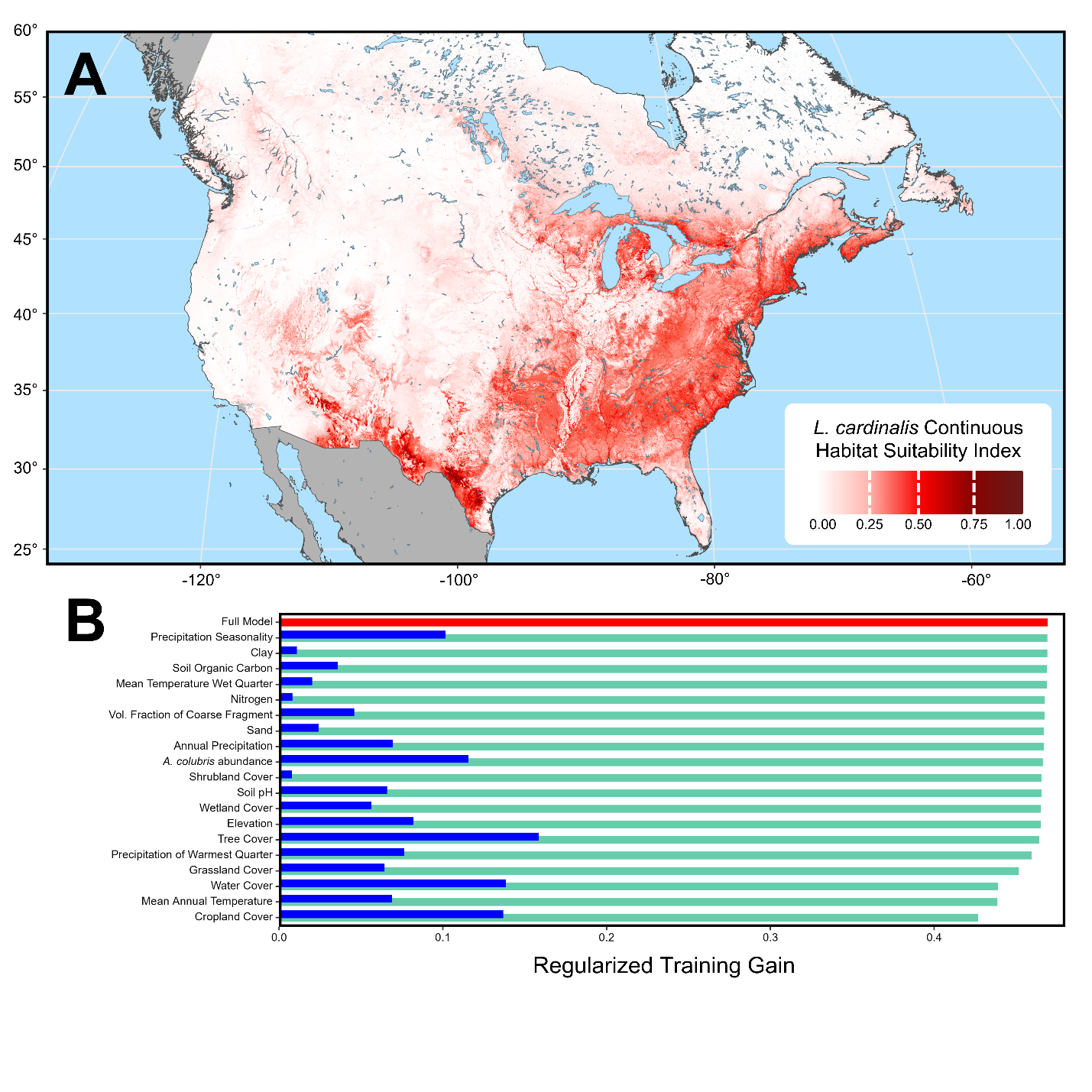


**Figure S6.** (A) Continuous habitat suitability map prediction for the supplementary MaxEnt model built at the spatial extent of all North America. Regions outside the model study area are in grey. Map is displayed using a Mollweide equal-area projection with a central meridian at –95º longitude. (B) Bar plot showing the jackknife test results for the supplementary MaxEnt model. The red bar shows the regularized training gain of the full model which includes all environmental variables. The turquoise bars show the training gain of models when a given variable was excluded from model building. The blue bars show the training gain of models where a given variable was used in isolation.


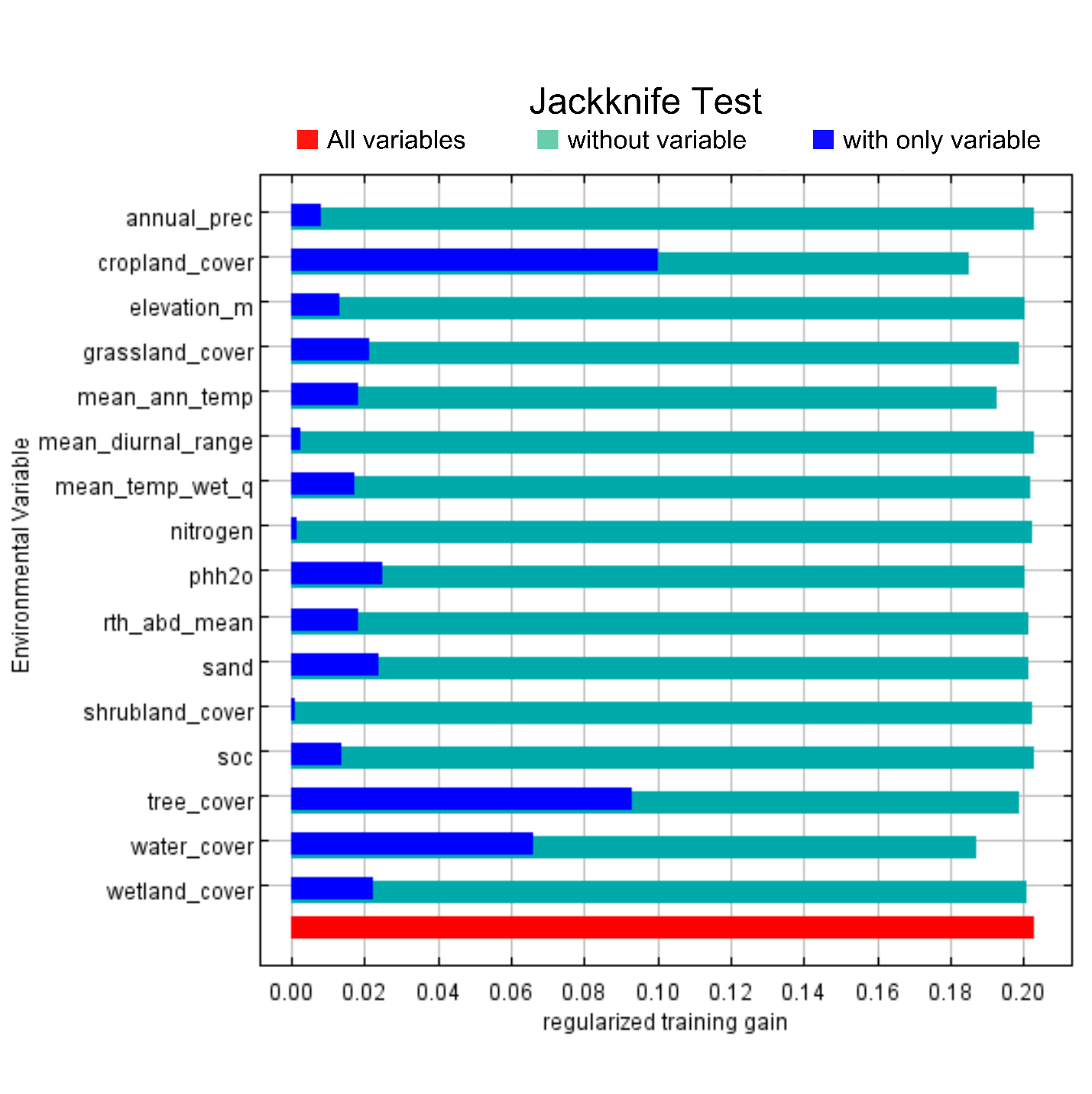


**Figure S7.** Bar plot showing the results of the jackknife tests for the supplementary MaxEnt model that used *Lobelia cardinalis* presence data from 2018 to 2022. The red bar shows the regularized training gain of the full model, which includes all environmental variables. The turquoise bars show the training gain of models when a given variable was excluded from model building. The blue bars show the training gain of models where a given variable was used in isolation.


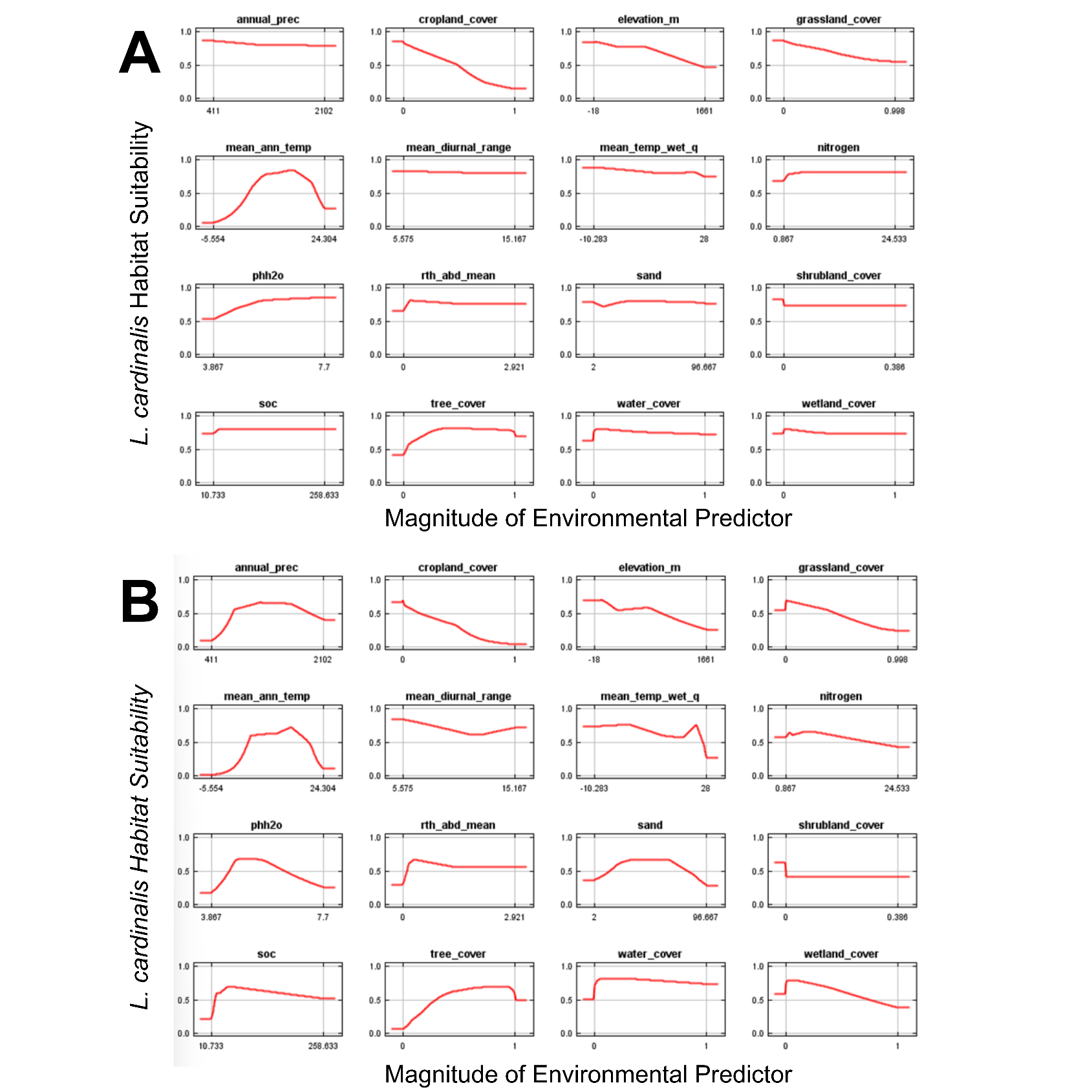


**Figure S8.** (A) The marginal and (B) variable-in-isolation response curves for the supplementary MaxEnt model that used *Lobelia cardinalis* presence data from 2018 to 2022.
