# Supplementary material for: The spatial distribution of a hummingbird‐pollinated plant is not strongly influenced by hummingbird abundance
Source: Am J Bot. 2025 Apr 26;112(5):e70034. doi: 10.1002/ajb2.70034 (PMC12094066; doi:10.1002/ajb2.70034)
Supplement: Supplementary file 2 — Appendix S2. Supplementary tables. Table S1. Lobelia species used in the species comparison analyses. Table S2. The results of the MaxEnt model optimization procedure performed with the R package ENMeval. Table S3. Linear model summary for the species comparison analysis focusing on between‐species variation in local flowering season abundance of Archilochus colubris. Table S4. Generalized linear model summary for the species comparison analysis focusing on between‐species variation in the ratio of Lobelia observation week to peak week of abundance of Archilochus colubris. Table S5. Model summary table of the Tukey contrasts from the species comparison analysis focusing on between‐species variation in the ratio of Lobelia observation week to peak week of abundance of Archilochus colubris. Table S6. Model evaluation metrics for the supplementary MaxEnt model, which used Lobelia cardinalis presence data from 2018 to 2022. Table S7. Percent contribution (PC) and permutation importance (PI) of each environmental variable for the supplementary MaxEnt model, which used Lobelia cardinalis presence data from 2018 to 2022. [file AJB2-112-e70034-s001.docx]

**Appendix S2.** Supplementary tables

**Table S1.** *Lobelia* species used in the species comparison analyses. The three rightmost columns show the mean iNaturalist observation date for each species and 2SD before and after this date. These dates were used as our metric for the flower period of each species.

| **Species** | **Number of observations** | **2SD Before** | **Average observation date** | **2SD After** |
| --- | --- | --- | --- | --- |
| *L.cardinalis* | 1747 | 1 July | 22 August | 13 October |
| *L. siphilitica* | 1378 | 22 July | 4 September | 19 October |
| *L. inflata* | 922 | 30 May | 14 August | 28 October |
| *L. spicata* | 275 | 10 May | 26 June | 13 August |
| *L. kalmii* | 176 | 30 June | 17 August | 3 October |
| *L. puberula* | 165 | 12 July | 15 September | 18 November |
| **Total** | **4663** |  |  |  |

**Table S2.** The results of the MaxEnt model optimization procedure performed with R package ENMeval. Below is a matrix showing the delta AICc value for each parameter combination (feature class [FC] and regularization multiplier). The parameter combination with a delta AICc value of zero (lowest AICc value) is bolded and marked with an asterisk.

|  | **Regularization multiplier** | | | | | | | | |
| --- | --- | --- | --- | --- | --- | --- | --- | --- | --- |
| **FC** | **1** | **1.5** | **2** | **2.5** | **3** | **3.5** | **4** | **4.5** | **5** |
| **L** | 415.37 | 421.22 | 421.14 | 426.09 | 432.17 | 439.50 | 447.97 | 457.92 | 468.94 |
| **LQ** | 96.57 | 123.88 | 160.18 | 193.02 | 222.53 | 249.41 | 283.28 | 318.93 | 337.57 |
| **H** | 79.14 | 92.09 | 77.97 | 67.87 | 72.33 | 68.34 | 86.22 | 70.37 | 108.90 |
| **LQH** | 48.07 | 22.51 | 16.56 | 14.50 | **0.00*** | 3.29 | 15.59 | 11.93 | 31.86 |

L, linear; LQ, linear and quadratic; H, hinge; LQH, linear, quadratic, and hinge.

**Table S3.** Linear model summary for the species comparison analysis focusing on between-species variation in local flowering season abundance of *Archilochus colubris*. Model coefficients indicate whether each species significantly differs from the reference group: *Lobelia cardinalis*.

| **Term** | **Estimate** | **SE** | ***P*** |
| --- | --- | --- | --- |
| (Intercept) | 0.3961 | 0.0047 | <0.001 |
| *Lobelia inflata* | -0.1019 | 0.0080 | <0.001 |
| *Lobelia kalmii* | -0.1895 | 0.0155 | 0.335 |
| *Lobelia puberula* | 0.0154 | 0.0160 | <0.001 |
| *Lobelia siphilitica* | -0.0333 | 0.0071 | <0.001 |
| *Lobelia spicata* | -0.0608 | 0.0127 | < 0.001 |

**Table S4.** Generalized linear model summary for the species comparison analysis focusing on between-species variation in the ratio of *Lobelia* observation week to peak week of abundance of *Archilochus* *colubris*. Model coefficients indicate that whether the ratio for a given species significantly differs from 1.

| **Term** | **Estimate** | **SE** | ***P*** |
| --- | --- | --- | --- |
| *Lobelia cardinalis* | -0.0156 | 0.0041 | <0.001 |
| *Lobelia inflata* | -0.0429 | 0.0057 | <0.001 |
| *Lobelia kalmii* | -0.0145 | 0.0134 | 0.277 |
| *Lobelia puberula* | 0.0758 | 0.0127 | <0.001 |
| *Lobelia siphilitica* | 0.0309 | 0.0045 | <0.001 |
| *Lobelia spicata* | -0.2893 | 0.0119 | <0.001 |

**Table S5.** Model summary table of the Tukey contrasts from the species comparison analysis focusing on between-species variation in the ratio of *Lobelia* observation week to peak week of abundance of *Archilochus colubris*.

| **Tukey contrast** | **Estimate** | **SE** | ***P*** |
| --- | --- | --- | --- |
| *L. cardinalis – L. inflata* | 1.028 | 0.0073 | <0.01 |
| *L. cardinalis – L. kalmii* | 0.999 | 0.0140 | 1.00 |
| *L. cardinalis – L. puberula* | 0.913 | 0.0122 | <0.001 |
| *L. cardinalis – L. siphilitica* | 0.955 | 0.0058 | <0.001 |
| *L. cardinalis – L. spicata* | 1.315 | 0.0166 | <0.001 |
| *L. inflata – L. kalmii* | 0.972 | 0.0141 | 0.373 |
| *L. inflata – L. puberula* | 0.888 | 0.0124 | <0.001 |
| *L. inflata – L. siphilitica* | 0.929 | 0.0068 | <0.001 |
| *L. inflata – L. spicata* | 1.280 | 0.0169 | <0.001 |
| *L. kalmii – L. puberula* | 9.14 | 0.0169 | <0.001 |
| *L. kalmii – L. siphilitica* | 9.56 | 0.0135 | 0.016 |
| *L. kalmii – L. spicata* | 1.316 | 0.0236 | <0.001 |
| *L. puberula – L. siphilitica* | 1.046 | 0.0141 | 0.011 |
| *L. puberula – L. spicata* | 1.441 | 0.0251 | <0.001 |
| *L. siphilitica – L. spicata* | 1.377 | 0.0175 | <0.001 |

**Table S6.** Model evaluation metrics for the supplementary MaxEnt model that used *Lobelia cardinalis* presence data from 2018 to 2022.

| **Presence data** | |
| --- | --- |
| 8324 presence points from GBIF – following data filtering: 5432 presence points | |
|  |  |
| **Model optimization –** lowest AICc combination | |
| Feature class: linear, quadratic, and hinge | Regularization multiplier: 1 |
|  |  |
| **Model cross validation** | |
| Training data: | 4345 points (80%) |
| Testing data: | 1087 points (20%) |
| Training AUC: | 0.779 |
| Training CBI: | 0.999 |
| Testing AUC: | 0.791 |
| Testing CBI: | 0.999 |
|  |  |
| **Final model evaluation** | |
| Training data: | 5432 points |
| Testing Data: | NA |
| Final AUC: | 0.782 |
| Final CBI: | ~1.00 |

**Table S7.** Percent contribution (PC) and permutation importance (PI) of each environmental variable for the supplementary MaxEnt model that used *Lobelia cardinalis* presence data from 2018 to 2022. Variables are ordered from highest to lowest percent contribution.

| **Value** | **PC (%)** | **PI (%)** |
| --- | --- | --- |
| Cropland cover | 29.1 | 34.4 |
| Water cover | 26.8 | 14.8 |
| Tree cover | 24.2 | 9.1 |
| Mean annual temperature | 6.0 | 14.4 |
| *Archilochus colubris* abundance | 2.9 | 1.0 |
| Elevation | 2.6 | 3.4 |
| Grassland cover | 2.3 | 11.0 |
| Wetland cover | 1.9 | 1.8 |
| Soil pH | 1.7 | 5.3 |
| Mean temperature wet quarter | 1.3 | 0.7 |
| Sand | 0.7 | 1.5 |
| Soil organic carbon | 0.4 | 0.3 |
| Nitrogen | 0.1 | 1.3 |
| Shrubland cover | 0.1 | 0.2 |
| Annual precipitation | 0.0 | 0.6 |
| Mean diurnal range | 0.0 | 0.1 |
